# Impact of Artificial Intelligence Assistance on Radiologist Performance in CT-Based Pulmonary Nodule Detection: A Systematic Review and Meta-Analysis of Detection Accuracy Studies

**DOI:** 10.3390/jimaging12100509

**Published:** 2026-10-14

**Authors:** Vinit Singh, Srihitha Gunda, Ali Shah, Joshua Mathew, Kapilraj Ravendran

**Affiliations:** 1 Faculty of Medicine, Medical University of Varna, 9002 Varna, Bulgariaalishahsuk@yahoo.co.uk (A.S.);; 2 Department of Orthopaedic Surgery, East and North Hertfordshire NHS Trust, Stevenage SG1 4AB, UK; k.ravendran@nhs.net

**Keywords:** artificial intelligence, computer-aided detection (CAD), pulmonary nodules, sensitivity, false positives

## Abstract

Introduction: Lung cancer remains a major cause of cancer-related death; prompt detection and monitoring of pulmonary nodules through computed tomography (CT) is critical to prevent mortality. CT interpretation is time-intensive and can be prone to fatigue-related errors. The application of artificial intelligence (AI) may aid in the enhanced detection of pulmonary nodules when used in conjunction with radiologists. Thus, possibly decreasing interpretation time and enhancing diagnostic reliability. However, studies have reported uncertainty regarding the extent to which true positives are always diagnosed, as studies have indicated an increase in false positive rates with AI-assisted pulmonary nodule detection. This systematic review and meta-analysis focuses on the impact of AI assistance on sensitivity and false positive rates in comparison to unaided reading. Methodology: Adhering to the PRISMA guidelines, an intensive literature search was carried out on Cochrane, PubMed, Medline and Embase. Research composed of studies that compared AI-assisted reading to unaided radiologists in the context of nodule detection, which also provided additional outcomes that were utilised in the secondary outcome analysis. ROBINS-1 tool was used to assess risk of bias and data quality. Risk ratios and risk differences with 95% CI were reported for dichotomous outcomes, whereas mean differences with 95% CI were utilised for continuous outcomes. Sensitivity analyses were performed for the imputation of missing standard deviations. Results: Seven studies reported mean sensitivity in either second reader (SR) mode or concurrent reader (CR) mode. Five of the included studies implemented second reader designs; AI-assisted sensitivity ranged from 38% to 85.66%, whereas unassisted reading sensitivity ranged from 20.9% to 73.46%. Additionally, three studies reported concurrent reading, with AI-assisted reading ranging from 80% to 84%, while unassisted reading varied from 64% to 72%. According to our research, AI-assisted reading had a significantly higher sensitivity (RR of 1.39; CI 95% 1.08–1.79; *p* = 0.01; I^2^ = 95%). Five studies reported false positives per scan. Four reported a numerical increase with AI assistance, but incomplete variance reporting and study heterogeneity precluded quantitative pooling. Reading time was increased under AI assistance as a second reader (MD 77.54; CI 95% −57.50 to 212.6, *p* = 0.132; I^2^ = 64.9%) but did not yield significance. Additionally, AI-assisted reading under concurrent reading did not yield statistical significance for reading time (MD –11.39; 95% CI −498.7 to 476.0, *p* = 0.816; I^2^ = 41.3%). Conclusions: AI-assisted reading significantly improves sensitivity for pulmonary nodule detection compared to unaided reading, but the trade-offs with false positives and workflow disruption remain uncertain. Substantial heterogeneity and the limited number of studies preclude definitive clinical recommendations. These findings should be considered exploratory, and the implementation of AI in lung cancer screening requires prospective validation.

## 1. Introduction

Early detection and monitoring of pulmonary nodules through low-dose computed tomography (CT) is crucial for reducing mortality, as lung cancer remains a major cause of cancer-related death worldwide [1,2]. Pulmonary nodules are often the earliest manifestations of lung cancer, and an early diagnosis is associated with improved prognosis for the patient [3]. Chest CT evaluation allows for detection of nodules, measurement of lesions, malignancy risk assessment, and management planning [4]. Moreover, interpretation of CT is a time-consuming process; it can be subject to diagnostic errors, interobserver and intraobserver variability, along with fatigue-related errors [5]. Detecting pulmonary nodules becomes especially challenging when the number of nodules present is not known, combined with time constraints [6]. Thus, these factors negatively affect and influence pulmonary nodule detection accuracy [4]. Transcending pulmonary nodule detection, artificial intelligence is increasingly integrated into wide-ranging radiology workflows, including image reconstruction and differential diagnosis generation. This wider adoption underscores the need to thoroughly investigate the diagnostic performance of AI in specific high-stakes tasks such as lung cancer screening.

The application of Artificial Intelligence-based tools may aid in lung cancer screening and could enhance the detection of pulmonary nodules when used in conjunction with radiologists compared to radiologists working alone. This was shown in a recent comparative study demonstrating AI assistance significantly increased detection from 64% to 77% among residents, while remaining largely unchanged for senior radiologists [7]. Even though AI assistance can aid in screening, it has been demonstrated that there is no significant difference in interpretation time for chest CT interpretation. Furthermore, there has been a greater frequency of follow-up low-dose CT recommendations, highlighting the potential downstream implications of improved detection [4].

These tools can assist in a series of tasks including image pre-processing, segmenting regions which are of interest, feature engineering and calculation, along with classification model development [8]. AI tools can automate lung nodule detection, classification and measurement, serving as an intervention to enhance and support radiologists’ assessment precision and diagnostic reliability [4].

While the diagnostic potential of AI algorithms for pulmonary nodule detection has been widely demonstrated in retrospective analyses and controlled reader studies, robust prospective data evaluating their integration into real-world lung cancer screening workflows remain limited. Some studies have documented that AI-assisted detection may reduce specificity when compared to a conventional radiologist’s interpretation, potentially increasing the frequency of follow-up imaging or additional diagnostic procedures and surgeries [4]. Other studies have reported low accuracy and even misdiagnosis. There is also uncertainty regarding the extent to which these tools may be helpful for highly experienced senior physicians, who have higher baseline diagnostic performance. AI-based tools may be more beneficial on a training level and for assisting less experienced doctors [1]. This highlights the need to evaluate to what extent AI assistance improves the performance of radiologists when detecting pulmonary nodules.

The aim of this systematic review and meta-analysis is to evaluate the impact of artificial intelligence assistance on a radiologist’s performance in detecting pulmonary nodules using computed tomography compared to a radiologist’s interpretation alone. It assesses outcomes using sensitivity and false-positive rates. Previous systematic reviews have evaluated AI-assisted nodule detection, but none have quantitatively synthesised sensitivity gains or false-positive rates specifically by reader paradigm or reader experience level [4,7]. Therefore, this paper provides a focused quantitative synthesis of these selected reader assistance outcomes, contributing additional granularity to the existing evidence base.

## 2. Methodology

### 2.1. Search Strategy

All authors collectively approved and developed a study protocol, with guidance and in accordance with the PRISMA (Preferred Reporting Items for Systematic Reviews and Meta-Analyses) standards [9]. Reviewer K.R. supervised the entire review process, including the literature search, data extraction and analysis. A risk of bias evaluation was conducted for each study, and discrepancies regarding bias evaluation or data interpretation were addressed through consensus discussions with reviewers (S.G., J.M.).

A systematic literature search was conducted using MEDLINE (via PUBMED), COCHRANE (including the Cochrane Central Register of Controlled Trials, Cochrane Database of Systematic Reviews, Database of Abstracts of Reviews of Effects, Cochrane Methodology Register, and National Health Service Economic Evaluation Database) and EMBASE. As per the search strategy, the following keywords were utilised: (“artificial intelligence” or “AI assistance” or “Deep Learning” or “computer-assisted” or “computer aided detection”) AND (“Radiologist” or “reader” or “observer” or “performance”) AND (“pulmonary nodule” or “lung nodule” or “nodule detection”) AND (“computed tomography” or “chest CT”) AND (“sensitivity” or “detection accuracy” or “observer study” or “reader study” or “false positive”). Additionally, to ensure comprehensive coverage of all relevant evidence, no date limitations were applied to the search strategy. The complete search syntax for each database is provided in Supplementary Material S1.

Following the database search, records were exported to reference management software. An automation tool was used to mark records as ineligible based on pre-defined criteria (e.g., non-English, non-human studies, irrelevant study design). Subsequently, two independent reviewers manually reviewed all marked records to confirm ineligibility, with discrepancies resolved by consensus.

This systematic review was guided by the following PICO structure: Population—radiologists interpreting chest CT scans for pulmonary nodule detection; Intervention—AI-assisted reading (either second-reader or concurrent-reader mode); Comparison—unaided radiologist interpretation; and Outcomes—sensitivity, false positives per scan, and reading time. The unit of analysis varied by outcome. For sensitivity, the unit was the nodule-reader observation (i.e., each reader’s detection status for each nodule). For false positives, the unit was the scan (false positives per scan). For reading time, the unit was the case (seconds per case).

The primary outcomes, sensitivity and false-positive rate per scan, were pre-specified in the registered protocol (PROSPERO: CRD420261349402). The additional outcomes (reading time, nodule size sensitivity, and reader experience sensitivity) were not pre-specified and were added as exploratory analyses. These findings should therefore be interpreted as hypothesis-generating rather than definitive.

### 2.2. Study Selection and Eligibility Criteria

This meta-analysis prioritised studies comparing AI-assisted reading with unassisted radiologist interpretation for pulmonary nodule detection on the same CT examination. The eligibility criteria included retrospective, prospective, and case-controlled studies incorporating within-reader comparisons, analysing the relevant outcomes of sensitivity or false positive rates. Moreover, non-human studies, papers reporting only standalone AI performance without data on the human comparator and non-English publications were excluded from consideration.

The primary outcome analysed was sensitivity for pulmonary nodule detection, under AI assistance in comparison to unassisted reading, and false positive rate per scan. Six outcomes were selected for additional comparison in the meta-analysis based on clinical relevance and availability of data: (1) second reader (SR) mode reading time, (2) concurrent reader (CR) mode reading time, (3) sensitivity for nodules ≤ 5 mm (exploratory, as these are not clinically reported in screening contexts), (4) sensitivity for nodules 6–10 mm, (5) sensitivity improvement for junior readers, and (6) sensitivity improvement for senior readers. All outcomes were selected to evaluate the impact of computer-aided detection (CAD) on diagnostic performance and workflow efficiency, with particular focus on nodule size and reader experience as potential modifiers. Additionally, baseline characteristics were extracted from each study, including the study design, total number of patients, nodules, readers and type of AI mode. Studies using both standard-dose CT and low-dose CT (LDCT) were eligible for inclusion, as both can be used in clinical practice for pulmonary nodule evaluation and our aim was to capture the full breadth of evidence on AI-assisted reading across different clinical contexts. For the size subgroup analyses, we attempted to extract data that corresponded to nodules ≤ 5 mm and nodules 6–10 mm. However, due to variations in reporting across the primary studies, the size intervals in the pooled analyses should be interpreted as approximate rather than exact. Specifically, Bogoni et al. reported sensitivity for nodules ≥ 3 mm and ≥5 mm; Kozuka et al. reported 3–6 mm and 6–10 mm; and Foti et al. reported 3 mm and 5 mm thresholds. These thresholds do not map precisely to our stated intervals of ≤5 mm and 6–10 mm. Consequently, we have labelled these analyses as exploratory and explicitly acknowledged the threshold inconsistencies in the Limitations section.

Where reported, we also extracted whether maximum intensity projection (MIP) reconstructions were used for nodule assessment. Only Foti et al. [10] explicitly reported MIP reconstructions for CAD display and multiplanar assessment. Roos et al. [11] reported that MIP display was available on the reading platform but did not specify it as the primary reconstruction for nodule assessment. The remaining five studies did not report MIP use. This is reported as ‘not reported’ rather than assumed absent. Similarly, no included study explicitly stated whether nodule size thresholds were determined on standard lung parenchymal window images or on MIP reconstructions. Foti et al. [10] used both lung window and multiplanar MIP for viewing but did not specify which was used for size measurement; Roos et al. [11] had MIP display available but did not specify it as the primary basis for size measurement. Because millimetric differences in measured nodule size can occur between lung window and MIP, the size subgroup analyses should be interpreted as approximate and hypothesis-generating.

For the purposes of this review, ‘AI-assisted reading’ was defined as any computer-aided detection (CAD) system or deep learning-based algorithm that provided automated identification or marking of potential pulmonary nodules for review by a radiologist, regardless of whether the system was used in second-reader or concurrent-reader mode. Studies using traditional feature-based CAD systems and modern deep learning platforms were both eligible for inclusion, provided they compared AI-assisted reading to unaided radiologist interpretation. ‘Junior readers’ were defined as radiologists-in-training, residents, or early-career radiologists with limited experience in chest CT interpretation, consistent with the definitions used in the included studies where available. ‘Senior readers’ were defined as fully qualified radiologists with extensive experience in chest CT interpretation. Furthermore, the classification of readers as ‘junior’ or ‘senior’ was based on the definitions used in the individual included studies, which varied across studies. For the reader experience subgroup analyses, we used the definitions of ‘junior’ and ‘senior’ readers as reported in the individual included studies. However, we acknowledge that these definitions varied across studies. For example, Kozuka et al. classified readers with 1 and 5 years of experience as ‘junior’, whereas other studies used 5 years as a threshold for ‘senior’ readers. Takaishi et al. reported a combined average for three readers with 2, 6, and 8 years of experience. Consequently, the pooled estimates for the junior reader subgroup should be interpreted as a general indication of CAD benefit among less experienced readers rather than a definitive comparison between junior and senior readers. These findings are exploratory.

The review protocol was registered at PROSPERO (CRD420261349402) on 24 March 2026. No deviations from the registered protocol occurred.

### 2.3. Data Extraction and Quality Assessment

Through predetermined protocols, two reviewers (V.S., S.G.) extracted data and a third reviewer (A.S.) resolved any discrepancies by facilitating consensus. The data extracted included study characteristics (e.g., author, year, design), number of patients, readers, nodules, reader experience, the AI type, all relevant outcomes measured (e.g., primary and secondary findings) and any implications. Furthermore, extraction of primary reported analysis data was done when studies reported sensitivity analyses that excluded outlier readers.

The Risk of Bias in Non-randomised Studies-of Interventions (ROBINS-1) tool was utilised by two authors (V.S., A.S.) independently to assess risk of bias and each study’s quality evaluation [12]. This methodological quality assessment was conducted given the comparative, non-randomised included study designs. The assessment encompassed confounding bias, bias in selection of participants into the study, bias in classification of interventions, bias due to deviation from intended interventions, bias due to missing data, bias in measurement of outcomes and bias in selection of the reported result. Additionally, the Risk of Bias Visualisation tool (ROBVIS) was adopted to summarise the quality evaluation graphically [13]. Moreover, all steps were performed under the guidance of K.R.

ROBINS-1 tool was selected because the included studies are non-randomised comparative reader performance studies evaluating an intervention (AI assistance) on radiologist performance, which aligns with ROBINS-1’s design for non-randomised studies of interventions. We therefore considered ROBINS-1 the most appropriate tool for assessing bias in these non-randomised intervention studies.

### 2.4. Statistical Analysis

This meta-analysis was performed in adherence to the Cochrane Collaboration’s guidelines and the Preferred Reporting Items for Systematic Reviews and Meta-Analysis (PRISMA) statement. The PRISMA 2020 and PRISMA-DTA checklists are provided as Supplementary Materials S3 and S4. Risk Ratios (RR) with 95% CI and Risk Difference (RD) with 95% CI were reported for dichotomous outcomes. Whereas, for continuous outcomes, mean differences (MD) with 95% CI were pooled. Furthermore, heterogeneity was analysed using the Cochrane Q test; a *p*-value of <0.10 would be considered statistically significant. Quantification of heterogeneity with I^2^ statistics categorises values of 0–40%, 30–60%, 50–90% and 75–100% as negligible, moderate, substantial, and considerable heterogeneity respectively. Additionally, a random-effects model was used to calculate pooled effect estimates, due to predicted clinical heterogeneity among the studies. Review Manager 5.4 (Nordic Cochrane Centre, The Cochrane Collaboration, Copenhagen, Denmark) was utilised for the primary statistical analysis.

For each of the six selected secondary outcomes, meta-analyses were performed using JASP (version 0.18.3; JASP Team, Amsterdam, The Netherlands) with random-effects models (DerSimonian–Laird method). Regarding reading time outcomes, the mean difference (MD) in seconds between CAD-assisted and unaided reading was calculated. For sensitivity outcomes, the risk difference (RD) was calculated as the absolute improvement in detection rate with CAD. Heterogeneity was assessed using the Q statistic and quantified with the I^2^ index, where I^2^ values of 25%, 50%, and 75% were considered low, moderate, and high heterogeneity, respectively. Forest plots were created displaying individual study effect sizes with 95% confidence intervals, pooled estimates with 95% confidence and prediction intervals, and study weights. Statistical significance was set at *p* < 0.05. Statistical significance was assessed using the t-distribution with k − 1 degrees of freedom, consistent with the random-effects model implemented in JASP. This approach accounts for uncertainty in estimating between-study variance (τ^2^) when the number of studies is small. Where the t-based *p*-value differs from the normal-approximation *p*-value derived from the confidence interval, the t-based *p*-value is reported as primary. A full list of the thresholds used in each included study and their mapping to the meta-analysis subgroups is provided in Supplementary Table S4.

Egger’s regression test and a funnel plot were employed to assess publication bias for the primary analysis.

Leave-one-out sensitivity analysis was performed for each secondary outcome to assess whether the pooled effect estimate was unduly influenced by any single study. This was done by sequentially removing one study at a time, recalculating the pooled effect, and observing whether the estimate changed substantially or crossed the null threshold.

A complete numerical audit was performed to ensure consistency across the manuscript, abstract, figures, and Supplementary Materials. The extracted dataset, including all raw data from the primary studies, is provided in Supplementary Material S2.

All included studies used paired multi-reader multi-case (MRMC) designs in which the same readers interpreted the same cases with and without AI assistance. This creates non-independence of observations due to within-reader and within-case clustering. An ideal meta-analysis would use paired effect estimates with MRMC-appropriate variance structures. However, none of the included studies reported the individual reader-case paired data or within-reader correlations required for such an analysis. Consequently, the confidence intervals reported here are likely too narrow and the statistical significance may be overstated. All findings should be interpreted as exploratory and hypothesis-generating.

False positives per scan were not pooled quantitatively because four of the five studies reporting this outcome did not report standard deviations. Imputation using coefficients of variation from a single study was considered but rejected, as it would assume comparable variance-to-mean relationships across different populations, CAD systems, readers, nodule prevalences, and false-positive definitions. False-positive findings are therefore summarised narratively.

All imputed SDs and raw data are provided in Supplementary Material S1.

### 2.5. Imputation of Missing Confidence Intervals

For studies that reported 95% confidence intervals instead of standard deviations (Bogoni et al.), standard errors were calculated using the formula: SE = (upper CI − lower CI)/(2 × 1.96) [14]. For one study (Foti et al.) that reported only the range of reading times, standard deviations were estimated as SD ≈ range/4 [10]. For sensitivity outcomes, the risk difference (RD) for each study was calculated as RD = Sensitivity_with_CAD − Sensitivity_without_CAD. Because all included studies used paired multi-reader multi-case (MRMC) designs, in which the same readers interpreted the same cases with and without AI assistance, the observations are not independent. The standard formula for independent proportions was therefore not appropriate. As none of the included studies reported the individual reader–case paired data or within-reader correlations required to compute paired standard errors, we were unable to derive MRMC-appropriate variance estimates. The standard errors used in the pooled analyses are therefore based on aggregate study-level data and do not account for within-reader or within-case clustering. Consequently, the confidence intervals reported here are likely too narrow and the statistical significance may be overstated. All findings should be interpreted as exploratory and hypothesis-generating. All transformed variables and calculations are provided in Supplementary Material S2.

## 3. Results

### 3.1. Literature Search

**Figure 1:**
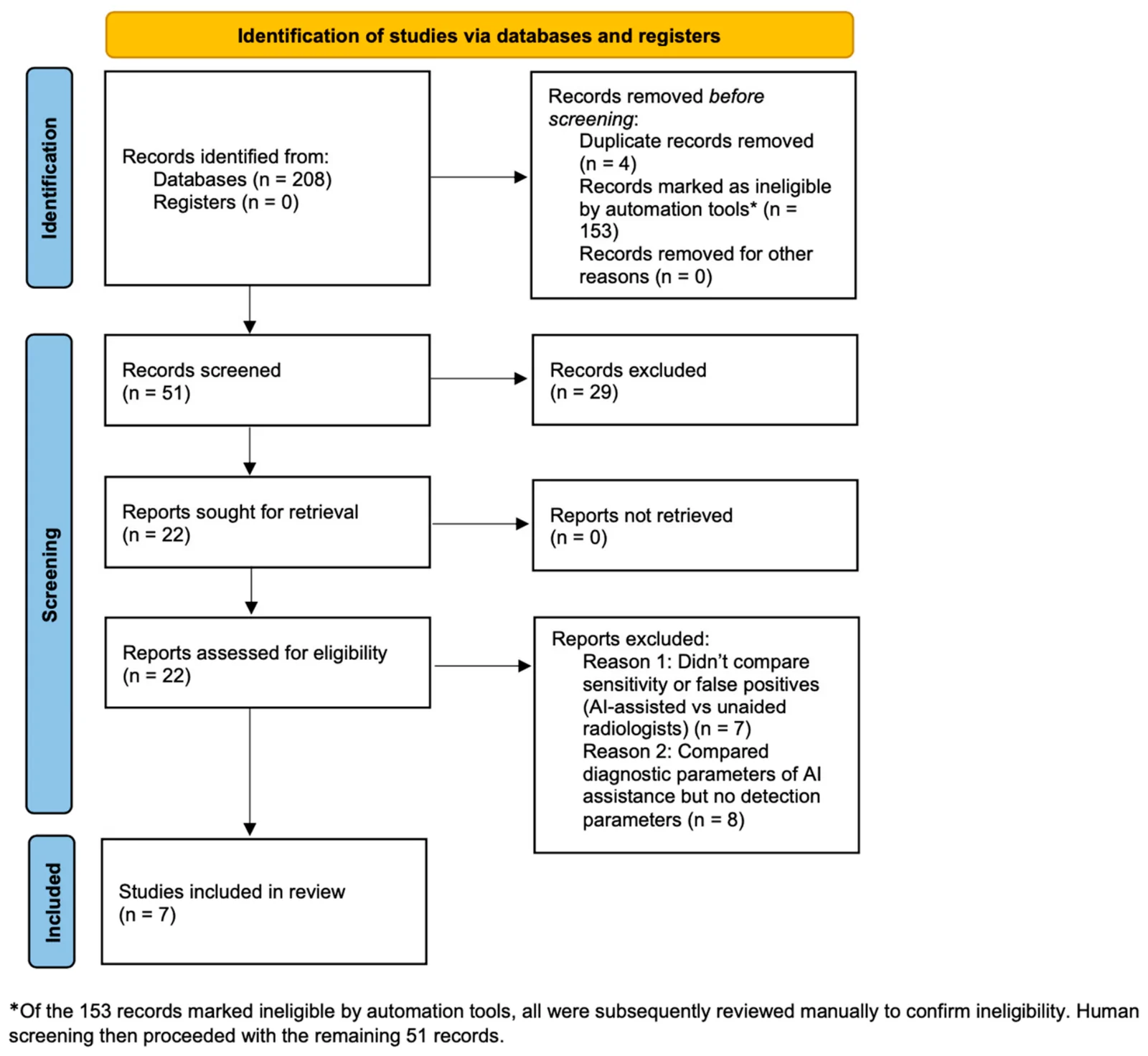
A PRISMA flow diagram to illustrate the process of literature search and selection.

**Figure 2:**
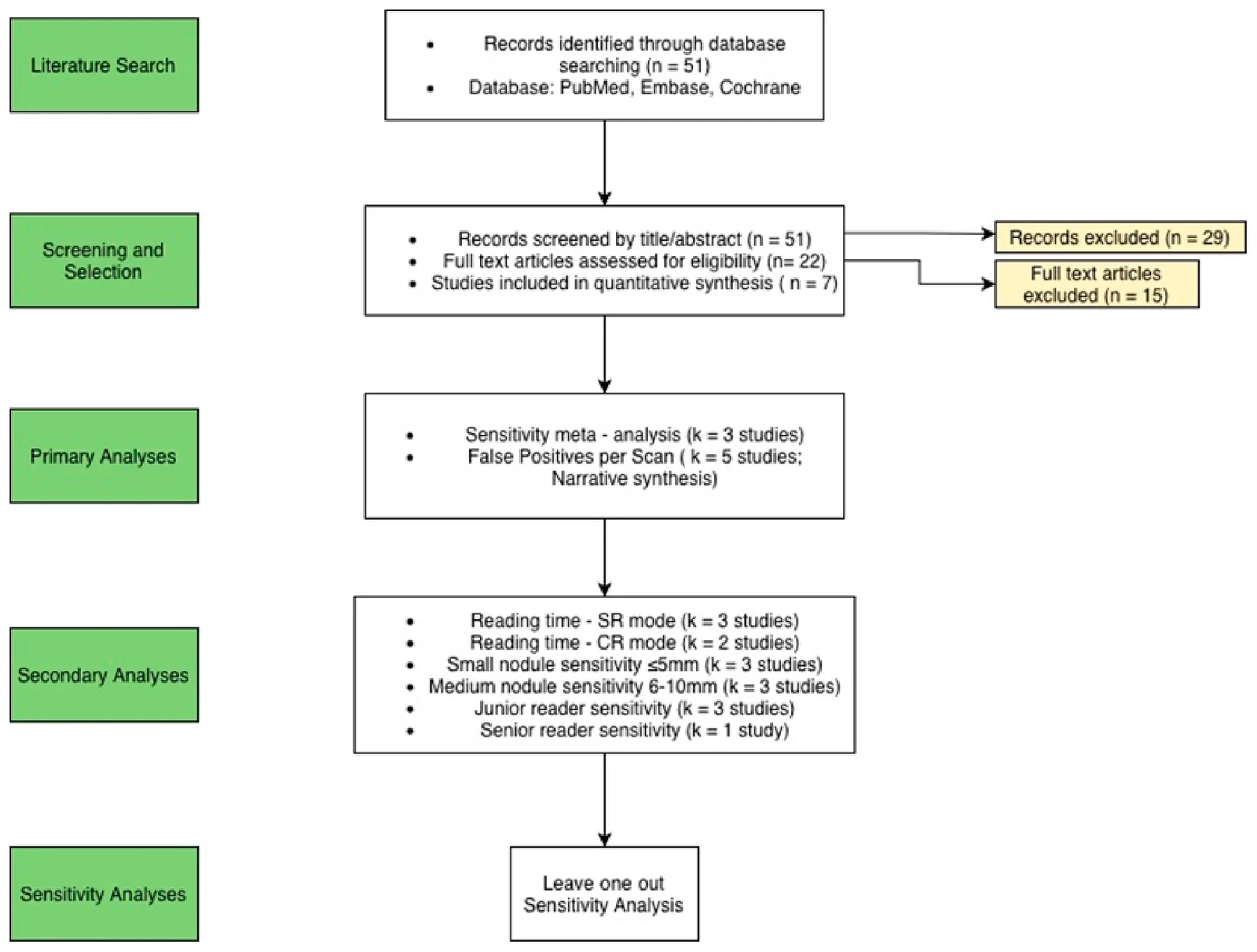
Graphical summary of the study workflow.

Following the removal of automated exclusions and duplicates, 51 records were identified through the systematic database search (full search strategies provided in Supplementary Material S1). Screening of titles and abstracts led to the exclusion of a further 29 records. Moreover, during full-text screening, 15 additional studies were deemed ineligible because they did not meet the inclusion criteria of the systematic review. The reasons for excluding full-text articles included: (1) no radiologist comparison (standalone AI only), (2) no extractable sensitivity or false positive data, (3) focus on diagnostic parameters rather than detection, and (4) non-English publications. A study-specific log of all 15 full-text exclusions, with reasons, is provided in Supplementary Table S3 and the numerical breakdown is provided in the PRISMA flow diagram (Figure 1). Therefore, 7 studies were included in the final meta-analysis, as illustrated by the PRISMA flow diagram (Figure 1) [9]. Figure 2 provides a graphical summary of the overall study workflow, including literature identification, study selection, primary analyses, secondary analyses, and sensitivity analyses.

The seven studies had a total of 805 patients who were enrolled in the retrospective multi-reader studies. These papers were published between 2010 and 2022 in the following countries: USA, Italy, China, Taiwan, Japan, and the United Kingdom. Furthermore, Table 1 summarises the key characteristics from each study and Table 2 summarises the technical characteristics of the included AI systems. In addition to this, a study-by-outcome data extraction table, including extracted numerators, denominators, reader mode, reader group, reference standard, nodule-size threshold, false-positive definition, and whether the study was included in quantitative or qualitative synthesis, is provided in Supplementary Table S1.

The primary meta-analysis focused on the utilisation of AI assistance in improving sensitivity and false-positive rates relative to unaided reading. The secondary analysis evaluated additional outcomes, including reading time difference in second-reader (SR) mode (seconds), reading time difference in concurrent-reader (CR) mode (seconds), sensitivity for nodules ≤ 5 mm or 3–6 mm (exploratory), sensitivity for nodules 6–10 mm, and sensitivity improvement for junior (<5 years experience) and senior readers (≥5 years experience).

### 3.2. Data Quality Assessment

**Figure 3:**
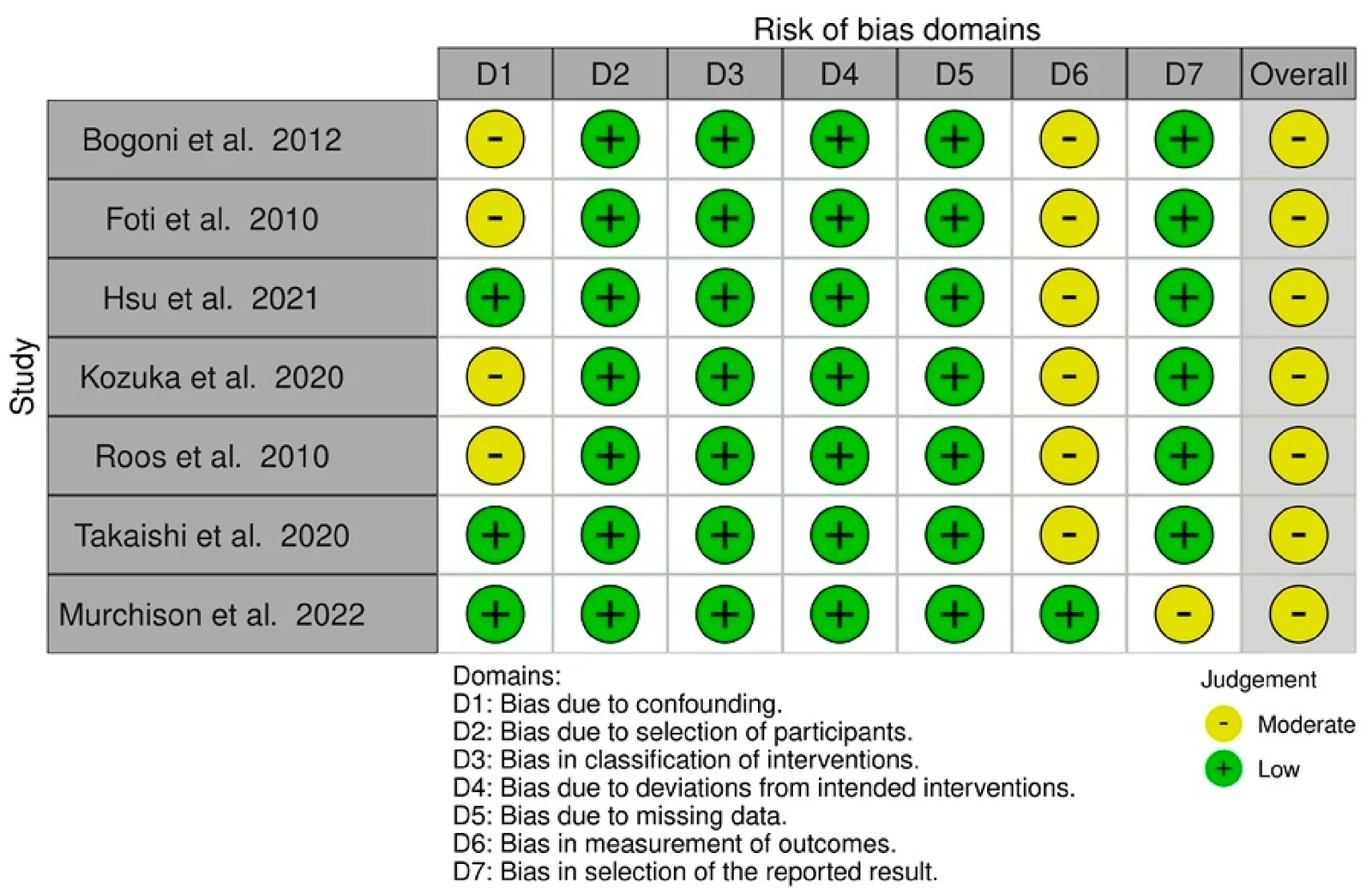
Data quality illustration through ROBVIS: risk of bias visualisation tool [13]. D1: Bias due to confounding; D2: Bias due to selection of participants; D3: Bias in classification of interventions; D4: Bias due to deviations from intended interventions; D5: Bias due to missing data; D6: Bias in measurement of outcomes; D7: Bias in selection of the reported result.

ROBINS-1 tool was utilised to evaluate the quality of the included studies and to assess risk of bias [12]. This tool was selected as it is designed for non-randomised studies of interventions, which aligns with the comparative reader performance design of the included studies. All the studies included are retrospective observational studies and were considered to have moderate risk of bias, due to bias in confounding, measurement of outcomes or selection of the reported result. Figure 3 illustrates the assessment of each study included in the meta-analysis.

### 3.3. Primary Analysis

#### 3.3.1. Sensitivity

The comparison of sensitivity for pulmonary nodule detection between AI-assisted reading and unaided radiologist reading was done through the extraction of true positive detections and the total number of nodule-reader observations for both groups. Sensitivity was calculated as TP/(TP + FN), where the total number of nodule-reader observations represented the denominator. Studies that did not report true positive counts were excluded from the primary forest plot. Only 3 studies met the inclusion criteria for quantitative synthesis: Bogoni et al. [14], Foti et al. [10], and Kozuka et al. [16]. Even though the remaining studies were excluded from the primary quantitative analysis due to incomplete reporting of raw data, they reported mean sensitivity. These outcomes were summarised qualitatively in either Table 3 or Table 4, due to methodological variation in reader paradigm (second reader or concurrent reader) across studies.

Both SR and CR modes were represented across the seven studies reporting mean sensitivity. Five of the included studies implemented second reader designs; unassisted radiologist sensitivity ranged from 20.9% to 73.64%, whereas AI-assisted sensitivity ranged from 38% to 85.66% (Table 3). Furthermore, three studies documented concurrent reading with unassisted reading varying from 64% to 72%, while AI-assisted reading varied from 80% to 84% (Table 4). Hsu et al. [15] reported both reader paradigms.

**Figure 4:**
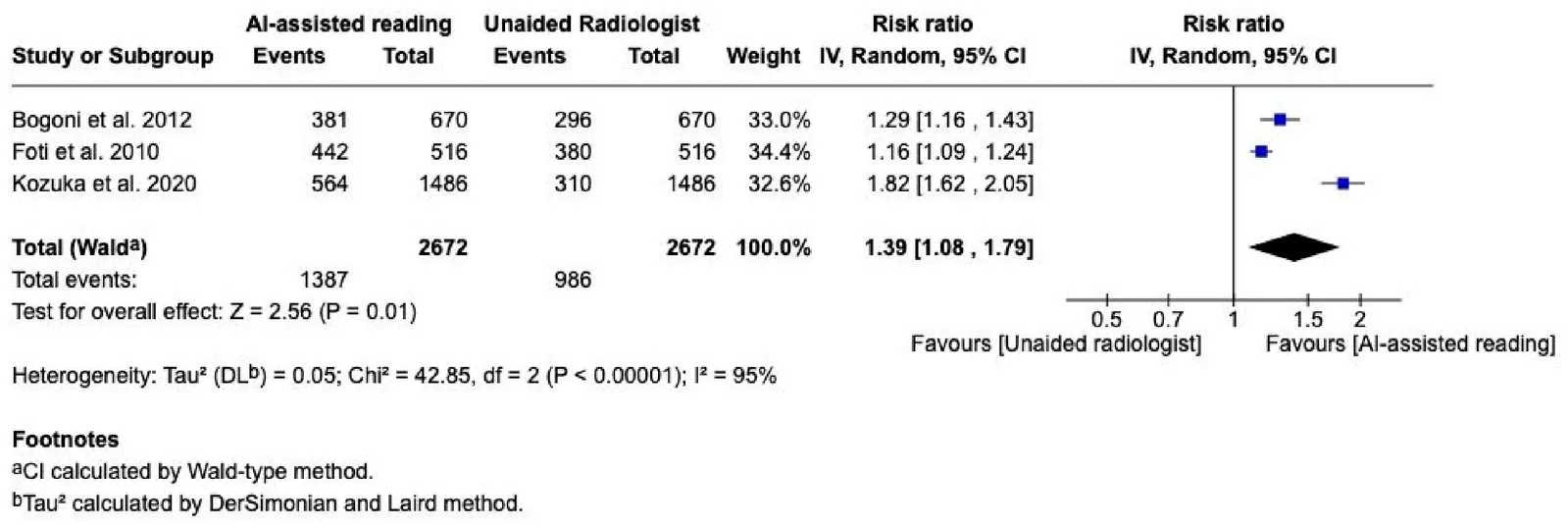
Shows that AI-assisted reading improves sensitivity compared with unaided reading.

**Figure 5:**
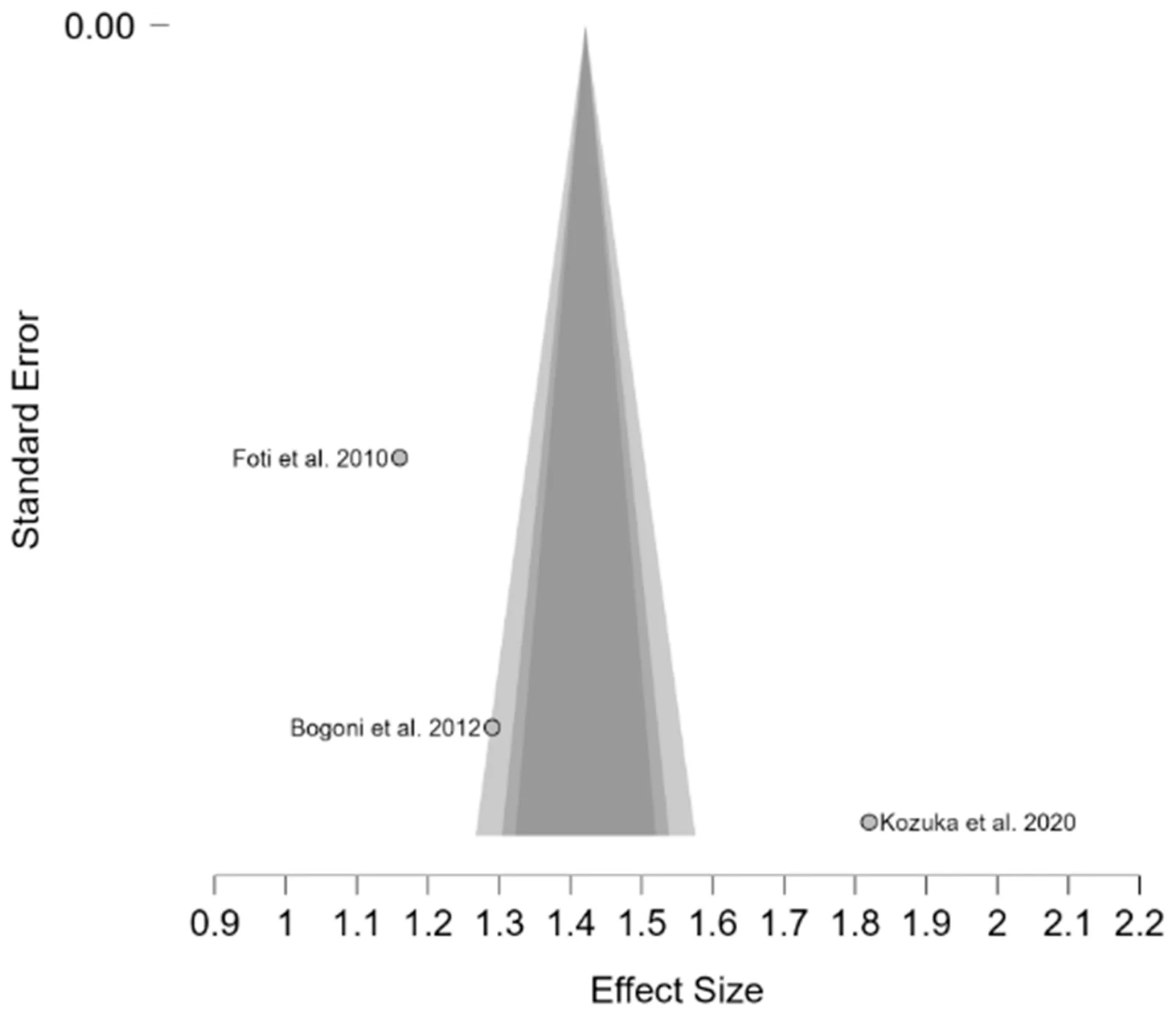
Demonstrates a funnel plot for the assessment of publication bias for sensitivity in AI-assisted reading and unaided reading. The study effect risk ratios are plotted against the standard errors. However, only 3 studies reported complete data; therefore, interpretation is limited.

Three studies, comprising both 2672 AI-assisted and unassisted readings, were quantitatively synthesised for the meta-analysis of relative sensitivity. AI-assisted reading was linked with significantly higher sensitivity, in comparison to unassisted reading. The forest plot (Figure 4) shows a pooled risk ratio (RR) of 1.39 (95% confidence interval [CI] 1.08–1.79, *p* = 0.01). Variability in study design, baseline reader performance, and AI systems was associated with the considerable heterogeneity observed (I^2^ = 95%). The funnel plot (Figure 5) did suggest possible asymmetry upon visual inspection for sensitivity. Moreover, Egger’s regression test was not significant (*p* = 0.336). However, only three studies were included due to missing data from the remaining papers, and the statistical power to detect true asymmetry is low. As a result, the publication bias assessment should be interpreted with appropriate caution.

**Figure 6:**
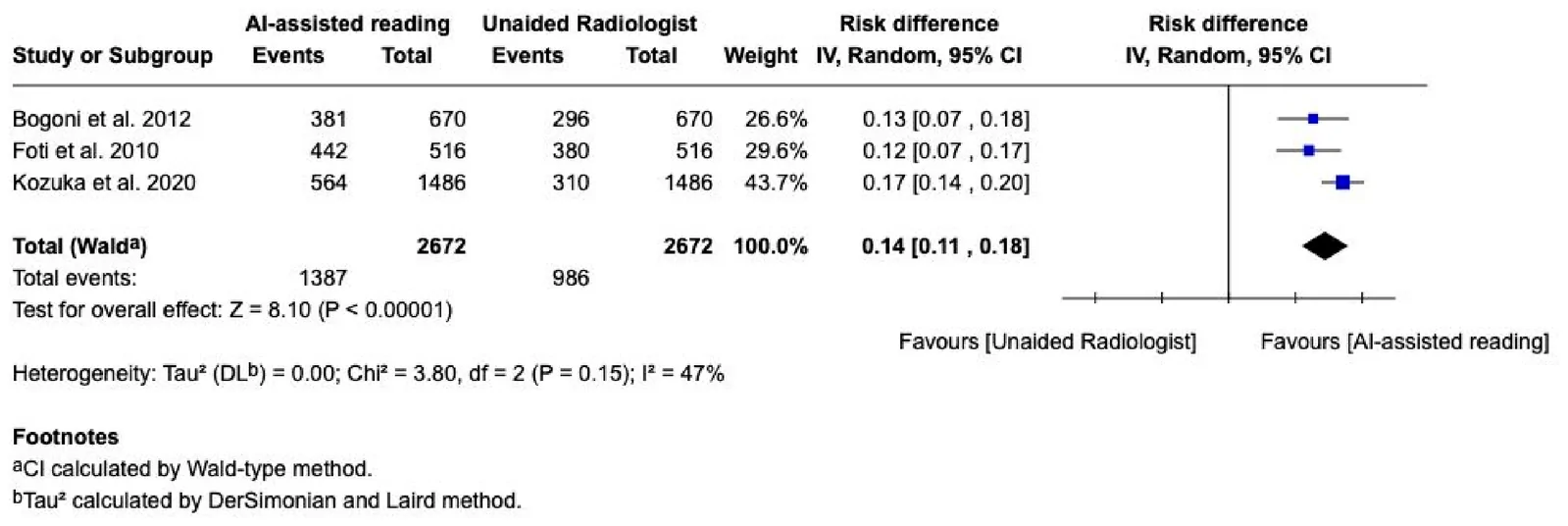
Forest plot comparing sensitivity risk differences.

Additionally, risk difference (RD) was also analysed for the same three included papers as an alternative effect measure (Figure 6). This was chosen to enable direct comparison with RR meta-analysis and to estimate the absolute gain in sensitivity attributed to AI-assisted reading, providing clinical intuition. The pooled RD was 0.14 (95% CI 0.11–0.18, *p* < 0.00001), emphasising an absolute enhancement in sensitivity of approximately 14 percentage points with AI assistance. Moreover, moderate heterogeneity was observed (I^2^ = 47%).

Due to the unavailability of individual reader-case paired data from the published studies, the following pooled estimates are based on aggregate study-level data. The confidence intervals do not account for within-study clustering by reader and case and should therefore be interpreted cautiously.

#### 3.3.2. False Positives

Five studies reported false positives per scan: Bogoni et al. [14], Foti et al. [10], Kozuka et al. [16], Roos et al. [11], and Murchison et al. [18]. Four of the five studies did not report standard deviations for this outcome. Because the studies also differed substantially in populations, CAD systems, reader experience, nodule prevalence, and definitions of false positives, these data were not pooled quantitatively. Imputation of standard deviations using coefficients of variation from a single study was considered but rejected, as this would assume comparable variance-to-mean relationships across heterogeneous settings.

Instead, false-positive findings are summarised narratively in Table 5. Four of five studies reported a numerical increase in false positives per scan with AI assistance, with increases ranging from 0.05 to 1.86 additional false positives per scan. One study (Foti et al. [10]) reported a small numerical decrease. The magnitude of change varied substantially across studies, and no formal publication-bias assessment was performed for this outcome because fewer than ten studies were available and no pooled estimate was calculated. These findings should be interpreted as directional and exploratory rather than as a quantitative estimate.

### 3.4. Secondary Outcomes

Six secondary outcomes were analysed: (1) SR reading time difference, (2) CR reading time difference, (3) small nodule sensitivity, (4) medium nodule sensitivity, (5) junior reader sensitivity, and (6) senior reader sensitivity. Due to the limited number of studies included for each outcome (k < 10 for all analyses), formal publication bias assessment using Egger’s regression test or funnel plot asymmetry was not performed, as such tests lack statistical power with fewer than ten studies and may produce misleading results.

#### 3.4.1. Reading Time—Second Reader (SR) Mode

Three studies reported reading time differences for SR mode: Bogoni et al. [14], Foti et al. [10], and Hsu et al. [15]. Roos et al. [11] was excluded from the quantitative synthesis because baseline reading times (582 s per case) were substantially longer than other studies (range 135–235 s), likely due to higher nodule burden (mean 9.5 nodules per scan vs. 2–4 in other studies), which would have introduced significant heterogeneity and biased the pooled estimate.

A random-effects meta-analysis was performed to evaluate the mean difference (MD) in reading time (seconds) between CAD-assisted SR reading and unaided reading, where a positive MD indicates longer reading time with CAD assistance.

**Figure 7:**
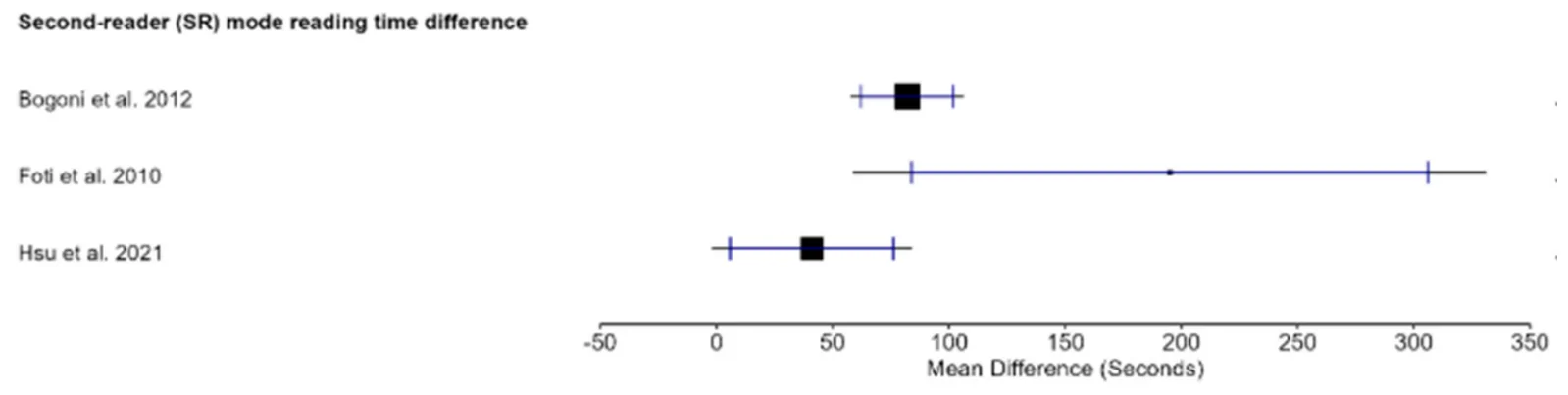
SR mode reading time difference: Pooled MD 77.54 s (95% CI -57.50 to 212.6, *p* = 0.132, I^2^ = 64.9%), indicating a non-statistically significant increase in reading time with CAD assistance in second-reader mode.

The pooled MD was 77.54 s (95% CI: −57.50 to 212.6, *p* = 0.132), indicating a non-statistically significant increase in reading time with CAD as a second reader (Figure 7). Substantial heterogeneity was observed (I^2^ = 64.9066). The Q-test for heterogeneity was not significant (Q(2) = 5.69, *p* = 0.058), though the *p*-value approached the conventional threshold. The *p*-value for this analysis is based on the t-distribution with k − 1 degrees of freedom, consistent with the random-effects model implemented in JASP. Where the t-based *p*-value differs from the normal-approximation *p*-value derived from the confidence interval, the t-based *p*-value is reported as primary.

Leave-one-out sensitivity analysis revealed that two studies (Bogoni et al. [14] and Hsu et al. [15]) were influential. Exclusion of Bogoni et al. [14] changed the pooled estimate to 95.94 s, while exclusion of Hsu et al. [15] changed the estimate to 62.41 s. Foti et al. [10] was not flagged as influential. These findings suggest that the pooled estimate is not robust, and the wide confidence interval crossing zero indicates that the observed increase in reading time with CAD assistance in SR mode is not statistically significant. This analysis is based on only three studies and should be considered exploratory.

#### 3.4.2. Reading Time—Concurrent Reader (CR) Mode

Two studies reported reading time differences for concurrent reader (CR) mode: Foti et al. [10] and Hsu et al. [15]. A random-effects meta-analysis was performed to evaluate the mean difference (MD) in reading time (seconds) between CAD-assisted and unaided reading, where a negative MD favours CAD-assisted reading (reduced reading time).

**Figure 8:**
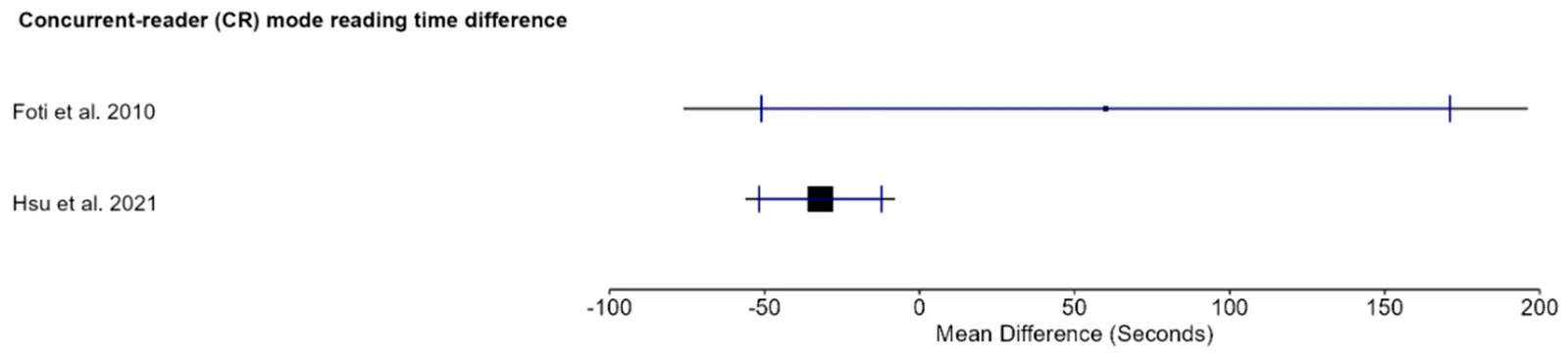
Forest plot of concurrent-reader (CR) mode reading time difference (seconds) between CAD-assisted and unaided reading. Negative values favour CAD-assisted reading (reduced time). Individual study mean differences are shown with 95% confidence intervals.

The pooled MD was −11.39 s (95% CI: −498.7 to 476.0, *p* = 0.816), indicating a non-statistically significant reduction in reading time with CAD assistance (Figure 8). Substantial heterogeneity was observed (τ^2^ = 1744, I^2^ = 41.3%). The Q-test for heterogeneity was not significant (Q(1) = 1.70, *p* = 0.192). The *p*-value for this analysis is based on the t-distribution with k − 1 degrees of freedom, consistent with the random-effects model implemented in JASP. Where the t-based *p*-value differs from the normal-approximation *p*-value derived from the confidence interval, the t-based *p*-value is reported as primary.

Leave-one-out sensitivity analysis revealed that both studies were flagged as influential. Exclusion of Foti et al. [10] changed the pooled estimate to 0.00 s, while exclusion of Hsu et al. [15] changed the estimate to 0.00 s. These findings suggest that the pooled estimate is not robust, and the extremely wide confidence interval crossing zero indicates that the observed reduction in reading time with CAD assistance in CR mode is not statistically reliable.

#### 3.4.3. Small Nodule Sensitivity (≤5 mm)

The size categories in this analysis vary across studies. Bogoni et al. reported sensitivity for nodules ≥ 3 mm; Kozuka et al. reported 3–6 mm; and Foti et al. reported 3 mm. These thresholds do not map precisely to the stated ‘≤5 mm’ interval. Therefore, these findings should be interpreted as a general indication of CAD performance across smaller nodules rather than a precise comparison of nodules ≤ 5 mm. Although nodules ≤ 5 mm are not routinely reported in lung cancer screening, we analysed this subgroup to evaluate CAD performance across all nodule sizes and to enable comparison with prior literature. These findings should therefore be considered exploratory.

Three studies reported sensitivity for small nodules (≤5 mm) with and without CAD assistance: Bogoni et al. [14], Foti et al. [10], and Kozuka et al. [16]. A random-effects meta-analysis was performed to evaluate the risk difference (RD) as the absolute improvement in detection rate with CAD assistance, where a positive RD favours CAD-assisted reading.

**Figure 9:**
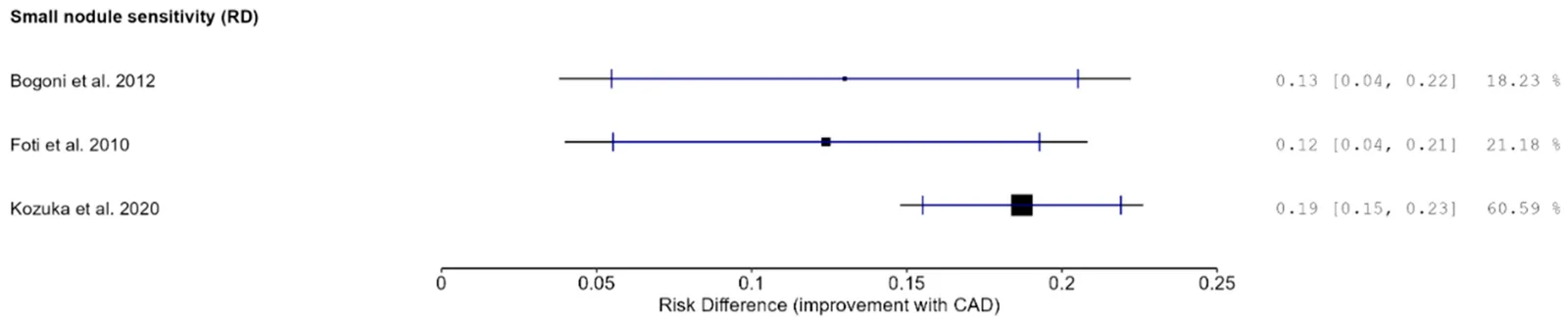
Small nodule sensitivity: Pooled RD 0.163 (95% CI 0.074 to 0.253, *p* = 0.016, I^2^ = 22.2%), indicating a significant absolute improvement of 16.3 percentage points with CAD assistance.

The pooled RD was 0.163 (95% CI: 0.074 to 0.253, *p* = 0.016), indicating a statistically significant absolute improvement of approximately 16.3 percentage points in small nodule sensitivity with CAD assistance (Figure 9). Heterogeneity was low (I^2^ = 22.2%). The Q-test for heterogeneity was not significant (Q(2) = 2.59, *p* = 0.274), suggesting that the observed variation across studies was compatible with chance.

This analysis combined non-equivalent thresholds across studies (see Supplementary Table S4) and should be interpreted as exploratory only.

Leave-one-out sensitivity analysis revealed that all three studies were flagged as influential. Exclusion of any single study changed the pooled estimate, but the direction and significance remained consistent. However, given that this analysis is based on only three studies, these findings should be considered exploratory rather than definitive. These findings indicate that the overall effect is robust despite the small number of studies.

In addition, no included study specified whether nodule size was measured on standard lung parenchymal window images or on MIP reconstructions. This is a further source of imprecision in the size intervals and reinforces the exploratory nature of these analyses.

#### 3.4.4. Medium Nodule Sensitivity (6–10 mm)

The size categories in this analysis vary across studies. Bogoni et al. reported sensitivity for nodules ≥ 5 mm; Kozuka et al. reported 6–10 mm; and Foti et al. reported 5 mm. These thresholds do not map precisely to the stated ‘6–10 mm’ interval. Therefore, these findings should be interpreted as a general indication of CAD performance across medium-sized nodules rather than a precise comparison of nodules 6–10 mm. Nodules in the 6–10 mm range are clinically significant and may require surveillance or diagnostic workup. Three studies reported sensitivity for medium nodules (6–10 mm) with and without CAD assistance: Bogoni et al. [14], Foti et al. [10], and Kozuka et al. [16]. A random-effects meta-analysis was performed to evaluate the risk difference (RD) as the absolute improvement in detection rate with CAD assistance, where a positive RD favours CAD-assisted reading.

**Figure 10:**
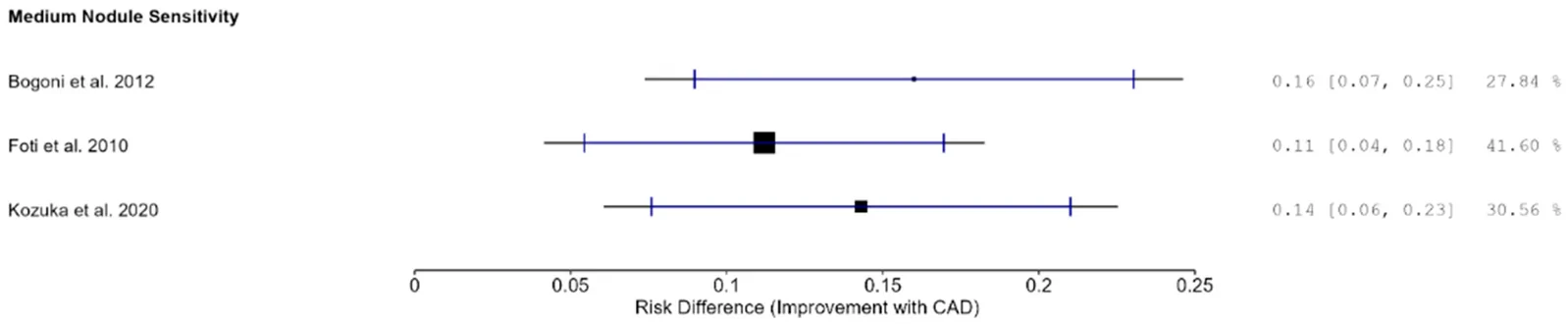
Medium nodule sensitivity: pooled RD 0.135 (95% CI 0.073 to 0.197, *p* = 0.011, I^2^ = 0%), indicating a significant absolute improvement of 13.5 percentage points with CAD assistance. The absence of heterogeneity (I^2^ = 0%) makes this one of the most robust findings among the secondary outcomes.

The pooled RD was 0.135 (95% CI: 0.073 to 0.197, *p* = 0.011), indicating a statistically significant absolute improvement of approximately 13.5 percentage points in medium nodule sensitivity with CAD assistance (Figure 10). Heterogeneity was zero (I^2^ = 0%, τ^2^ = 0.000), representing one of the most consistent findings across all secondary outcomes. The Q-test for heterogeneity was not significant (Q(2) = 0.77, *p* = 0.681), confirming that the observed variation was well within the range expected by chance. This analysis combined non-equivalent thresholds across studies (see Supplementary Table S4) and should be interpreted as exploratory only.

Leave-one-out sensitivity analysis revealed that all three studies were flagged as influential. Exclusion of any single study changed the pooled estimate, but the direction and significance remained consistent. However, given that this analysis is based on only three studies, these findings should be considered exploratory despite the consistent direction of effect and absence of heterogeneity. With only three studies, the estimate of heterogeneity is imprecise, and the absence of heterogeneity should not be interpreted as evidence of a homogeneous effect across all settings.

In addition, no included study specified whether nodule size was measured on standard lung parenchymal window images or on MIP reconstructions. This is a further source of imprecision in the size intervals and reinforces the exploratory nature of these analyses.

#### 3.4.5. Junior Reader Sensitivity

Three studies reported sensitivity improvement with CAD assistance specifically for junior or inexperienced readers: Hsu et al. [15], Kozuka et al. [16], and Takaishi et al. [17]. A random-effects meta-analysis was performed to evaluate the risk difference (RD) as the absolute improvement in detection rate with CAD assistance for junior readers, where a positive RD favours CAD-assisted reading. Junior readers, defined as radiologists-in-training, residents, or early-career radiologists with limited experience in chest CT interpretation, were analysed separately from senior readers. The specific definitions used in each study varied (e.g., Hsu et al. [15] defined juniors as those with 1–2 years of CT experience and seniors with 5 years of experience; Kozuka et al. [16] considered readers with 1 and 5 years as less experienced).

**Figure 11:**
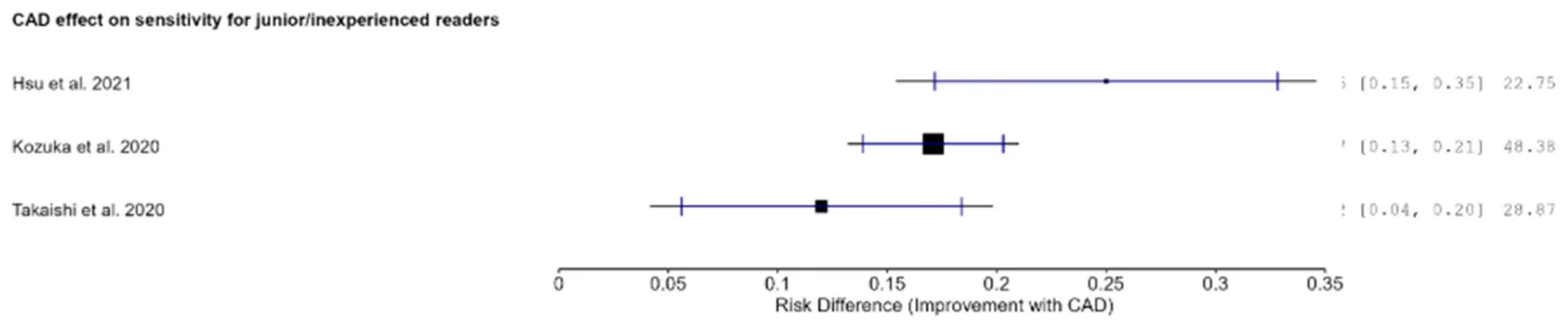
Forest plot of junior reader sensitivity risk difference between CAD-assisted and unaided reading. Positive values favour CAD assistance (improved sensitivity). Pooled RD 0.174 (95% CI 0.033 to 0.316, *p* = 0.034, I^2^ = 52.5%), indicating a significant absolute improvement of 17.4% percentage points with CAD assistance.

The pooled RD was 0.174 (95% CI: 0.033 to 0.316, *p* = 0.034), indicating a statistically significant absolute improvement of approximately 17.4 percentage points in sensitivity for the junior reader subgroup (Figure 11). Heterogeneity was moderate (I^2^ = 52.5%, τ^2^ = 0.001). The Q-test for heterogeneity was not significant (Q(2) = 4.22, *p* = 0.121). However, the definition of ‘junior’ readers varied across studies. Kozuka et al. included a reader with 5 years of experience, and Takaishi et al. reported a combined average for readers with 2, 6, and 8 years of experience. Therefore, this finding should be interpreted as a general indicator of CAD benefit among less experienced readers rather than a definitive comparison between junior and senior readers. This analysis is based on heterogeneous definitions of ‘junior’ readers across studies (see Supplementary Table S4) and should be interpreted as exploratory only.

Leave-one-out sensitivity analysis revealed that Takaishi et al. [17] was flagged as influential. Exclusion of this study resulted in a reduction in heterogeneity (τ^2^ = 0.001) and a change in the pooled estimate. Hsu et al. [15] and Kozuka et al. [16] were not flagged as influential. These findings suggest that while the overall effect is statistically significant, the moderate heterogeneity and influential study indicate that the magnitude of benefit should be interpreted cautiously. This analysis is based on only three studies and should be considered exploratory.

#### 3.4.6. Senior Reader Sensitivity

Only one study reported sensitivity improvement with AI assistance specifically for senior or experienced readers: Hsu et al. [15]. Senior readers were defined as fully qualified radiologists with extensive experience in chest CT interpretation. Hsu et al. [15] included senior readers with 5, 10, and 25 years of experience. In this study, AI assistance improved sensitivity for senior readers by an absolute risk difference of 0.11 (11 percentage points). However, with only a single study available, a meta-analysis could not be performed, and no pooled estimate or heterogeneity statistics could be calculated. These findings should be interpreted cautiously, and further studies are needed to confirm the benefit of AI assistance for senior readers.

Due to the limited availability of senior reader data and the inconsistent definitions of ‘junior’ readers across studies, a direct comparison between junior and senior readers could not be reliably performed. The apparent difference in benefit between junior and senior readers (17.4% vs. 11%) should be interpreted with caution and should not be taken as evidence that junior readers benefit more than senior readers, as this comparison is based on non-standardised definitions.”

## 4. Discussion

A pulmonary nodule is defined as a “round opacity that is moderately well marginated and no greater than 3 cm in diameter” [19]. The importance of early detection of suspicious pulmonary nodules cannot be overstated, as it is one of the early manifestations of lung cancer, a very common, aggressive and highly heterogeneous disease. The use of CT as the primary imaging modality for detection, characterisation and follow-up of pulmonary nodules with either neoplastic or benign aetiology has been considered a gold standard for many years [20,21,22]. CT is considered superior to conventional radiographic techniques as it produces cross-sectional 3D images, and its higher contrast resolution allows tissues to be differentiated easily. In modern clinical practice, the use of MDCT (multi-detector computed tomography) has become commonplace as it allows nodules that are less than 1 cm to be detected [23]. The superiority of CT was evidenced by Henschke et al. in the Early Cancer Action Project (ELCAP), which reported a 3 times greater detection rate of non-calcified nodules with low-dose CT compared with conventional chest radiography [24].

The potential role of AI-based CAD in detecting pulmonary nodules has been supported by several pilot studies, which demonstrated that CAD modules can identify overlooked pulmonary nodules that radiologists may miss, offering an additional layer of diagnostic assurance [25]. An older study by Sahiner et al. proposed that CAD’s most significant diagnostic gain was in sub-centimetre nodules (3–4 mm), where its use as a second reader provides a vital safety net for “sub-visual” findings [26]. While architectural shifts in CAD and deep learning systems have vastly improved their ability to distinguish true nodules from complex anatomical noise, such as vascular intersections and pleural surfaces, the clinical utility of this sensitivity has been redefined [27]. Contemporary clinical screening guidelines, such as ACR Lung-RADS v2022, have shifted the paradigm to prioritise the detection of actionable nodules > 6 mm, as smaller micronodules have an exceedingly low risk of malignancy and do not alter clinical patient management [28]. Furthermore, while two-stage detection frameworks such as the model proposed by Li et al. (2018) that pairs a broad candidate screening algorithm with a deep learning filter attempt to mitigate false positives, achieving clinical parity remains a challenge [29]. Indeed, such architectures can still yield significantly higher false-positive rates per scan compared to traditional radiologist double reading, underscoring that the ‘false-positive hurdle’ remains a key barrier to seamless clinical integration. These studies support the conclusion that modern CAD integration significantly reduces the rate of missed nodules, providing a robust, objective standard for diagnostic detection in high-volume screening environments. However, whether this translates to reduced interval cancers or improved patient outcomes requires prospective evaluation.

Transcending pulmonary nodule detection, studies have shown the growing utility of large language models (LLMs) throughout diverse radiology applications. LLMs such as ChatGPT-4o have been shown to potentially enhance the diagnostic performance of general radiologists in cardiac imaging, indicating their potential as diagnostic support tools [30]. Additionally, LLMs have shown promise for the selection of appropriate imaging modalities for a range of clinical scenarios, as supportive tools in clinical practice [31]. In the context of lung cancer screening, LLMs have shown promising performance on Lung-RADS-related questions, with certain models achieving a 96% accuracy rate [32]. These parallel developments highlight the broader paradigm shift toward AI-assisted clinical decision support across radiology, reinforcing the relevance of our focused evaluation of AI-assisted detection accuracy for pulmonary nodules.

More recent model versions have also shown promise in lung cancer-specific workflows. In a real-world evaluation of 150 lung cancer multidisciplinary team (MDT) cases, Gemini-2.5-Pro and GPT-5 both generated treatment recommendations broadly concordant with expert MDT plans, with Gemini-2.5-Pro achieving the highest overall balance (micro-F1 0.7603; accuracy 0.8647) when provided with complete clinical information [33]. GPT-5 demonstrated higher recall (0.9132 with structured input) but lower precision, reflecting a tendency toward broader recommendations requiring clinician filtering. These findings suggest that current-generation LLMs may be useful as adjuncts in lung cancer decision support, although the authors emphasised that their role should remain assistive rather than autonomous, with human oversight required for complex cases. Prospective, version-controlled evaluation of these models within lung cancer screening and nodule management workflows therefore remains an important direction for future research.

Our systematic review and meta-analysis seek to synergise the data gathered from various studies on the efficacy of CAD assistance in detecting pulmonary nodules. While the studies we referenced do seek to provide insight into specific reader modes and sensitivity metrics, such as Takaishi et al. [17] and Kozuka et al. [16], our objective is to overcome the limitation of small sample sizes and localised demographics. We intend to harmonise disparate findings to provide a robust and statistically significant estimate of CAD’s impact on sensitivity, false-positive rate and reading time. Ultimately, our meta-analysis highlights how AI performs under different deployment strategies, specifically comparing its utility as a concurrent reader versus an independent second reader in CT lung cancer screening.

Recent studies published after our search date, including those from 2026, are cited in the introduction and discussion to provide context on the evolving landscape of AI-assisted nodule detection. However, these studies were not included in our meta-analysis as they either did not compare sensitivity or false positive rates, focused on diagnostic parameters rather than detection accuracy, or did not report the sensitivity data required for our quantitative or qualitative synthesis.

Our primary analysis observed an improvement in sensitivity with AI assistance; these findings were consistent across the literature. However, this evidence relates to detection performance under controlled, retrospective conditions and does not directly demonstrate improvements in patient-level outcomes such as cancer diagnosis or survival. Lan et al. (2022) established that AI-assisted reading enhanced detection of ‘error-prone’ lung nodules that could be missed, such as small sizes, central locations, and ground-glass appearances [34]. Moreover, these findings aligned with the Health Technology Assessment (HTA) report by Geppert et al. [35], which reported that AI may increase cancer and nodule detection but could consequently lead to an unnecessary increase in CT surveillance [35]. Similarly, Ewals et al. (2023) examined AI-assisted nodule detection and reported improvements in sensitivity, which was consistent with our findings [36]. However, it was noted that direct and comparative evidence was not found on cost-effectiveness and clinical outcomes [35]. Furthermore, our results revealed an RR of 1.39 (*p* = 0.01), which aligns with the range of sensitivity improvements (+5 to +20) reported by Geppert et al. [37] for clinically significant nodules. However, the term ‘actionable nodules’ as used by Geppert et al. [37] refers to clinically significant nodules, whereas our subgroup analyses were based on size thresholds of ≤5 mm and 6–10 mm. Therefore, our findings and those of Geppert et al. (2024) are not directly comparable, and readers should interpret our results in the context of our size-based definitions [37]. In the context of the primary analysis, these pooled estimates should be interpreted with caution, as the included studies are multi-reader designs with clustering by reader and case that could not be accounted for in our meta-analysis due to unavailable paired data. Therefore, these findings should be considered descriptive and hypothesis-generating rather than definitive.

An important observation from our synthesis is the marked difference in the range of AI-assisted sensitivity between reader paradigms. In second reader (SR) mode, sensitivity ranged widely from 38% to 85.66%, whereas in concurrent-reader (CR) mode the range was notably narrower at 80–84%. This discrepancy likely reflects the greater heterogeneity among SR studies in terms of AI system type (ranging from traditional feature-based CAD to machine learning-based platforms like ClearRead CT, and deep learning-based systems), reader experience (ranging from 1 to 25 years), nodule burden, and study period (2010 to 2021). In contrast, the CR studies were more relatively homogeneous, as all employed more contemporary AI-based systems in more recent years (2020–2022). This observation underscores that the benefit of AI assistance is not solely determined by the reader paradigm, but is also influenced by the specific AI system, reader expertise, case mix, and temporal context. Future research should aim to standardise these factors to enable more meaningful comparisons across reader paradigms.

A trend towards an increase in false positive detections with AI-assisted reading was quantified by Lo et al. (2018), demonstrating an FP of 0.17 to 0.28 per study case for all studies [38]. Moreover, these findings are not unique to modern deep learning systems, with Katase et al. (2022) reporting 3.1/7.25 FPs per scan, possibly resulting in an increase in effort and reading time for radiologists [39]. Across the five studies in our review that reported false positives per scan, four reported a numerical increase with AI assistance, with increases ranging from 0.05 to 1.86 additional false positives per scan, while one study reported a small numerical decrease. Because of incomplete variance reporting and substantial heterogeneity in populations, CAD systems, and false-positive definitions, these data were not pooled. The consistency of direction across most studies suggests a potential trade-off between improved sensitivity and increased false positives, but the magnitude of this trade-off remains uncertain.

Elevated false-positive rates may lead to additional invasive diagnostic procedures, heightened patient anxiety, and increased healthcare costs. A randomised control trial, which was a Feasibility study for a National Lung Screening Trial, reported that 7% of participants with a false-positive low-dose CT examination underwent an invasive procedure as a result [40]. Moreover, a randomised Danish Lung Cancer Screening Trial (DLCST) concluded that receiving a false-positive result in lung cancer screening was linked to psychosocial consequences [41]. Furthermore, a study documented greater medical care expenditures associated with false positives [42].

The impact of CAD assistance on reading time differed between reader modes. In second reader (SR) mode, where radiologists reviewed CAD marks after their initial interpretation, reading time increased by a pooled mean of 77.54 s per case (*p* = 0.132); this was not statistically significant. This finding aligns with the expected workflow cost of a sequential “read then review” paradigm, as radiologists must allocate additional time to evaluate CAD prompts, dismiss false positives, and confirm true positives [25]. In contrast, concurrent-reader (CR) mode demonstrated a non-significant reduction in reading time of 11.39 s (*p* = 0.816), suggesting that displaying CAD results simultaneously with the original CT images may streamline interpretation. However, the heterogeneity (I^2^ = 41.3%) and influential study diagnostics indicate that the CR time saving is not robust across all settings.

Beyond reading time, the choice between SR and CR modes has implications for radiologist training and skill maintenance. In SR mode, radiologists interpret scans independently before reviewing AI marks, preserving their own diagnostic skills. In CR mode, however, radiologists cannot ‘unsee’ the AI marks presented at the start, which partly explains why CR mode is faster. A scoping review found that AI can impair physicians’ performance or reduce opportunities for skill maintenance [43]. Furthermore, a study comparing AI timing strategies found that secondary mode was appreciated for minimising bias and aiding training, whereas concurrent mode was preferred for its balance between efficacy and reader control [44]. Radiology trainees who rely on AI during the early formative years of clinical education may fail to develop the foundational reasoning skills that safe, independent practice requires; this is referred to as “never skilling”. The regular and exclusive use of CR mode may also risk de-skilling, where radiologists lose the ability to detect nodules without AI assistance. This is particularly concerning for trainees who are still developing their interpretive skills. A balanced approach that uses CR mode selectively while periodically using SR mode or AI-free reading, thus may help preserve diagnostic competency while reducing the risk of “never skilling” and “de-skilling” [45].

These findings have important practical implications. Reading time varied considerably with case difficulty and nodule burden. Roos et al. [11] reported substantially longer reading times (582 s per case) in a cohort with high nodule prevalence (mean 9.5 nodules per scan), compared to other studies where nodule burden was lower (2–4 per scan). Similarly, Hsu et al. [15] found that junior readers required more time (183 s per case) than senior readers (128 s per case) when reading unaided. These findings suggest that the impact of CAD on reading time may be influenced by baseline case complexity and reader experience, factors that should be considered when interpreting pooled estimates.

While SR mode may be preferred in high-stakes scenarios where maximising sensitivity is paramount (e.g., baseline lung cancer screening), its time penalty could limit throughput in high-volume clinical practices. Conversely, CR mode offers potential workflow efficiency but may require optimisation of CAD display interfaces to avoid visual clutter and ensure that the time benefit is reliably achieved [37]. The substantial heterogeneity observed across studies highlights that the effect of CAD on reading time depends on contextual factors, including nodule prevalence, CT dose protocol, reader experience, and CAD system characteristics. However, given the limited number of studies (k ≤ 3 for SR mode and k = 2 for CR mode), these reading time findings should be considered preliminary and exploratory rather than definitive workflow conclusions. In addition, the included studies varied substantially in case complexity and nodule burden; for instance, Roos et al. [11] reported a mean of 9.5 nodules per scan compared to 2–4 nodules per scan in other studies, likely contributing to the wide variation in baseline reading times (ranging from 135 to 582 s). This substantial clinical and methodological variability limits the reliability and generalizability of the reading time pooled estimates, which should be considered preliminary hypothesis-generating findings rather than definitive workflow conclusions.

CAD assistance was consistently associated with improved sensitivity across different size categories. For medium-sized nodules (approximated by the 6–10 mm interval), the pooled RD was 0.135 (95% CI: 0.073 to 0.197, *p* = 0.011). For smaller nodules (approximated by the ≤5 mm interval), the pooled RD was 0.163 (95% CI: 0.074 to 0.253, *p* = 0.016). However, the thresholds used to define these categories varied across studies (see Table 6 footnote), and the intervals should therefore be interpreted as approximate. While the improvement for 6–10 mm nodules is clinically relevant, the observed benefit for smaller nodules should be considered exploratory, as these nodules are not routinely reported in lung cancer screening contexts. However, this finding should be considered exploratory, as these nodules do not typically trigger follow-up or intervention in screening programmes. Nevertheless, this observation may have relevance in diagnostic CT settings where all nodules are reported, or in secondary analyses of screening data where the natural history of small nodules is being investigated.

Notably, the medium nodule (6–10 mm) analysis showed zero heterogeneity (I^2^ = 0%), representing the most consistent finding across all secondary outcomes. However, given that this analysis is based on only three studies, these findings should be considered exploratory. The consistency observed suggests a potential benefit that warrants further investigation in larger studies, rather than a definitive, predictable effect. This consistency suggests that CAD provides a predictable, reliable benefit for nodules in the 6–10 mm range, regardless of study design, CT protocol, or reader population. Medium nodules are large enough to be consistently identified by CAD algorithms but still carry diagnostic uncertainty that benefits from computer-aided confirmation. In contrast, small nodule sensitivity exhibited low heterogeneity (I^2^ = 22.2%), reflecting less variability in the detection of sub-5 mm nodules across different CAD systems and imaging conditions.

From a research perspective, the differential improvement by nodule size suggests that CAD demonstrates the greatest diagnostic gain for the smallest, most elusive nodules. Even though these are not clinically significant in screening, they still provide a meaningful benefit for clinically significant nodules (6–10 mm). Screening programmes that implement CAD may therefore expect the greatest improvement in detection sensitivity for small nodules, which are most challenging to identify by unaided reading [26]. However, the clinical significance of detecting nodules < 6 mm remains uncertain in screening contexts, and improved detection in reader studies does not directly translate to patient benefit. Whether this improved detection translates to reduced interval cancers or improved survival requires prospective evaluation.

While our analysis of nodules ≤ 5 mm showed improved detection with CAD, it is important to contextualise these findings within current clinical practice. Per Lung-RADS guidelines, nodules < 6 mm are not routinely reported in lung cancer screening as they have a very low probability of malignancy [46]. Therefore, the clinical significance of improved detection for these very small nodules is uncertain. These data may, however, be relevant for diagnostic CT settings or research contexts where characterisation of all nodule sizes is of interest.

CAD assistance was associated with improved sensitivity for the junior reader subgroup (pooled RD 0.174; 95% CI: 0.033 to 0.316; *p* = 0.034). For senior readers, only one study (Hsu et al. [15]) reported data, showing an improvement of 0.11 (11 percentage points). However, the definitions of ‘junior’ readers varied across the included studies. Kozuka et al. classified a reader with 5 years of experience as ‘junior’, while Takaishi et al. reported a combined average for readers with 2, 6, and 8 years of experience. Therefore, the apparent difference in benefit between junior and senior readers should be interpreted with caution and is not directly comparable. The finding suggests a potential benefit of CAD for less experienced readers that warrants further investigation in studies with standardised reader experience definitions. The findings suggest a potential benefit that requires confirmation in larger, prospective studies. This suggests that CAD may help bridge the experience gap, bringing less experienced readers closer to the performance level of their senior colleagues.

The moderate heterogeneity for junior readers (I^2^ = 52.5%) indicates that the magnitude of benefit varies across studies, potentially due to differences in training level definitions (e.g., 1 year vs. 4 years of experience), case mix complexity, or CAD system usability. Only one study reported senior reader outcomes, precluding assessment of heterogeneity or meta-analysis for this subgroup.

These findings have important workforce implications. In training settings, lung cancer screening CTs are typically read jointly by residents and senior radiologists as part of the educational process. Our exploratory analysis suggests that CAD may offer educational benefits by helping trainees identify nodules they might otherwise miss, potentially accelerating their learning curve. However, this remains speculative and requires prospective evaluation. Integrating CAD into this workflow could reduce missed nodules, though this remains a hypothesis based on limited exploratory data that requires prospective evaluation. The time cost of SR mode (77.54 s) may be less acceptable for senior readers, who already interpret high volumes of cases; CR mode may offer a better trade-off for experienced radiologists, providing efficiency without compromising diagnostic accuracy.

AI as a primary triage tool was not evaluated in this study but represents an emerging implementation strategy. Jiang et al. [47] evaluated the feasibility of an AI-first reader workflow, which concluded that only 1.9% of participants required further radiologist review. Moreover, the authors stated the impact of perifissural nodules on the maximum achievable workload reduction was negligible. The choice of implementation strategy (AI as a primary triage tool, SR mode or CR mode) should be tailored to the clinical setting, reader experience, and available resources, if these findings are confirmed in future research. Our stratified analyses suggest that SR mode may be better suited for scenarios where maximising sensitivity is paramount, while CR mode may offer workflow efficiency benefits. However, these findings are based on very limited data (k ≤ 3 for most analyses) and should be considered exploratory; they require prospective validation before any definitive clinical recommendations can be made.

Our findings have important implications for the application of AI-assisted detection within current pulmonary nodule management frameworks, especially the Lung CT Screening Reporting and Data System (Lung-RADS) [46]. Lung-RADS is used to methodise and standardise the management of screen-detected pulmonary nodules. Adams et al. [48] showed that Lung-RADS used in conjunction with a machine learning classifier greatly improved timing of diagnosis and reduced follow-up across cohorts. It was concluded that when compared to Lung-RADS, a computer-assisted diagnosis software enhanced incidental detection of lung nodules and risk classification on screening chest CT scans [46]. However, our analysis observed a concurrent increase in false positives, raising a parallel concern. This trade-off between improved sensitivity and increased FPs is particularly relevant in the context of Lung-RADS, as category assignment directly determines the timing and intensity of follow-up. Furthermore, recent evidence suggests that AI can serve as a safety net for incidental identification of pulmonary nodules, with 30% of clinically significant incidental nodules that would have been missed brought to clinical attention by an AI software [49]. Overall, these findings underscore the need for prospective evaluation of AI-assisted reading within Lung-RADS-based screening programmes to determine whether the observed improvements in detection sensitivity translate to meaningful changes in nodule management and patient outcomes.

### Limitations

Several limitations should be considered when interpreting our findings. First, and most importantly, all included studies used paired multi-reader multi-case (MRMC) designs, in which the same readers interpreted the same cases both with and without AI assistance. This creates non-independence of observations due to within-reader and within-case clustering, which violates the independence assumption of standard meta-analytic models. An ideal meta-analysis would use paired effect estimates with MRMC-appropriate variance structures (e.g., Obuchowski-Rockette or bootstrap methods). However, none of the included studies reported the individual reader-case paired data or within-reader correlations required for such an analysis. Consequently, the confidence intervals reported here are likely too narrow and the statistical significance may be overstated. All findings should therefore be interpreted as exploratory and hypothesis-generating rather than definitive. Related to this, statistical significance was assessed using the t-distribution with k − 1 degrees of freedom, consistent with the random-effects model implemented in JASP. This approach accounts for uncertainty in estimating between-study variance (τ^2^) when the number of studies is small. Where the t-based *p*-value differs from the normal-approximation *p*-value derived from the confidence interval, the t-based *p*-value is reported as primary. We recommend that future primary studies report individual reader-case data or paired effect estimates with appropriate variances to facilitate more robust meta-analyses. Second, all included studies were retrospective in design, which may introduce selection bias and limit external validity to prospective screening populations. The included studies also varied in their target populations and clinical intended uses, ranging from lung cancer screening cohorts to cancer patients undergoing staging to general clinical populations. This heterogeneity may limit the generalisability of our findings to specific clinical contexts. However, the consistent direction of effect across these diverse settings, with AI consistently improving sensitivity, suggests that the benefit of AI assistance may be generalisable across different patient populations. Third, the reference standards varied across studies; most used expert consensus or expert panel review to define the ground truth, while others relied on radiology reports with or without AI confirmation. Fourth, reader experience levels varied considerably across studies, with inconsistent definitions of ‘junior’ and ‘senior’ readers. Thus, introducing potential verification bias. Fifth, the AI systems themselves were heterogeneous, spanning traditional feature-based CAD systems and modern deep learning platforms, which prevents direct comparison of algorithm types and limits the generalisability of our pooled estimates. Sixth, while we stratified sensitivity and reading time results by reader paradigm, we could not stratify false-positive rates or other secondary outcomes by reader mode due to insufficient data across studies. This limits our ability to draw definitive conclusions about the comparative effectiveness of SR versus CR modes across all outcomes. Seventh, the included studies used heterogeneous imaging protocols. While Hsu et al. [15] included both standard-dose and low-dose CT images, most other studies used standard-dose CT in clinical or suspected-cancer populations. Low-dose CT has become a standard imaging modality for lung cancer screening [50]. The inclusion of standard-dose CT studies may have influenced baseline detection rates and the magnitude of AI-associated benefit. However, as our primary aim was to evaluate the comparative performance of AI-assisted versus unaided reading across diverse clinical settings, we considered the inclusion of both imaging protocols appropriate. Nevertheless, this heterogeneity should be considered when interpreting the pooled estimates.

Beyond these study-level limitations described above, several data-related limitations affected our quantitative synthesis. Firstly, only three studies provided the raw data necessary for the quantitative sensitivity meta-analysis, substantially limiting the strength and generalizability of this pooled estimate. The remaining studies, while directionally consistent, could only be assessed qualitatively. Secondly, we were unable to perform a quantitative meta-analysis of false positives per scan because four of the five studies reporting this outcome did not report standard deviations. We considered imputing standard deviations using coefficients of variation from a single study, but rejected this approach because it would assume comparable variance-to-mean relationships across different populations, CAD systems, readers, nodule prevalences, and false-positive definitions. False-positive findings are therefore presented narratively. Future studies should report standard deviations or confidence intervals for false-positive rates to enable quantitative synthesis. Thirdly, the substantial heterogeneity observed in our primary sensitivity analysis (I^2^ = 95%) warrants careful interpretation. Potential sources include marked differences in reader experience (ranging from 1 to 25 years), CT acquisition protocols (standard-dose versus low-dose), nodule prevalence and burden across study populations, and the underlying AI or CAD system architecture (traditional feature-based versus deep learning). For example, the wide variation in unassisted sensitivity across studies (ranging from 20.9% to 73.64%) likely reflects these differences; Kozuka et al. reported the lowest unassisted sensitivity (20.9%) with the least experienced readers (1 and 5 years), whereas Foti et al. reported the highest unassisted sensitivity (73.64%) with more experienced readers (3–11 years) and potentially less challenging case mix. While the direction of effect was consistent across all studies, with AI assistance consistently improving sensitivity, the magnitude of benefit varied considerably. This suggests that the benefit of AI assistance is context-dependent and may be influenced by case mix, reader expertise, and technical implementation. To account for this, a random-effects model was utilised. Fourthly, exploration of sources of heterogeneity was not possible due to incomplete reporting of data; future studies should report confidence intervals or SDs for FPs to facilitate precise meta-analyses. Fifthly, publication bias was measured using Egger’s regression test and a funnel plot; while the results did not indicate publication bias, the small number of studies precludes definitive conclusions. Finally, all studies were assessed to have a moderate risk of bias, primarily due to confounding and measurement outcomes. While these retrospective studies represent the best available evidence, it warrants cautious interpretation of the pooled effect estimates. To minimise confounding, we restricted our review to studies in which the same radiologists interpreted scans with and without assistance, thus strengthening the internal validity of our pooled estimates. Leave-one-out sensitivity analyses confirmed that the significant findings for SR reading time, small nodule sensitivity, and medium nodule sensitivity were robust to the exclusion of any individual study. However, the CR reading time result was not robust, with two studies flagged as influential, indicating that this finding should be interpreted cautiously.

The nodule-size subgroup analyses combined non-equivalent thresholds across studies. For example, the ‘≤5 mm’ analysis included nodules ≥ 3 mm (Bogoni), 3–6 mm (Kozuka, Hsu), ≤4 mm and >4 to <6 mm (Hu), and a 3 mm threshold (Foti). The ‘6–10 mm’ analysis included ≥5 mm (Bogoni), 6–10 mm (Kozuka, Hsu), 6–8 mm and >8 mm (Hu), and a 5 mm threshold (Foti). This may introduce heterogeneity and limit the interpretability of these findings. Future studies should adopt standardised size thresholds to facilitate pooling. A full list of the thresholds used in each included study is provided in Supplementary Table S4. Consequently, these findings should be interpreted as a general indication of CAD performance across different nodule sizes rather than a precise comparison of nodules ≤ 5 mm versus 6–10 mm. We have explicitly noted these threshold inconsistencies in Table 6 and throughout Section 3.

The reader experience subgroup analysis was also limited by inconsistent definitions across studies. Kozuka et al. classified readers with 1 and 5 years of experience as ‘junior’, whereas other studies used 5 years as a threshold for ‘senior’ readers. Takaishi et al. reported a combined average for three readers with 2, 6, and 8 years of experience, rather than reporting individual reader-level data. Consequently, the pooled estimate for the junior reader subgroup is based on heterogeneous definitions and should be interpreted as a general indication of CAD benefit among less experienced readers rather than a definitive comparison between junior and senior readers. A full list of the definitions used in each included study is provided in Supplementary Table S4.

MIP reconstruction use was inconsistently reported across studies. Only Foti et al. [10] explicitly reported MIP for CAD display. This may have influenced nodule conspicuity and detection thresholds, and future studies should report reconstruction parameters in detail. In addition, none of the included studies specified whether nodule size was measured on standard lung parenchymal window images or on MIP reconstructions. Foti et al. [10] used both lung window and multiplanar MIP for viewing but did not specify which was used for the final size measurement, and Roos et al. [11] had MIP display available without specifying it as the basis for size measurement. Because millimetric differences in measured nodule size can occur between these approaches, this may have shifted nodules across the ≤5 mm and 6–10 mm thresholds and represents an additional source of error in the size subgroup analyses.

Regarding risk of bias assessment, we acknowledge that alternative tools such as QUADAS-2 exist for diagnostic accuracy studies [51]. We also acknowledge that ROBINS-I does not fully capture certain aspects specific to reader-performance studies, such as reading order, washout, blinding, and recall effects. However, given that our included studies were comparative reader performance studies evaluating an intervention (AI assistance) rather than diagnostic test accuracy studies, we considered the ROBINS-1 tool appropriate for assessing bias in non-randomised studies of interventions. We have also performed a supplementary QUADAS-C assessment for reader-performance-specific items (Supplementary Table S2), which showed consistent findings.

To improve transparency and reproducibility, future AI-assisted reader studies should adhere to established reporting guidelines such as TRIPOD+AI, STARD-AI, and CLAIM [52,53,54]. Based on the limitations identified in our meta-analysis, we specifically recommend that future studies report raw contingency tables (true positives, false positives, false negatives), provide standard deviations or confidence intervals for continuous outcomes, clearly define reader experience using standardised categories, describe AI system characteristics in detail, and specify the reader paradigm.

## 5. Conclusions

AI-assisted reading improved sensitivity for pulmonary nodule detection compared with unaided reading, with a similar direction of effect in both second-reader (SR) and concurrent-reader (CR) modes, although a direct statistical comparison between paradigms was not possible and false-positive rates could not be stratified by reader paradigm. Across the five studies reporting false positives per scan, four reported a numerical increase with AI assistance, but incomplete variance reporting and study heterogeneity precluded quantitative pooling, and the magnitude of this trade-off remains uncertain. Reading time increased in SR mode (not significant), with no significant difference in CR mode. Exploratory secondary analyses suggested possible benefit for medium-sized nodules (approximately 6–10 mm) and for less experienced readers, but nodule-size thresholds and reader-experience definitions varied across studies, and improved detection of nodules ≤ 5 mm is of uncertain clinical significance as these are not routinely reported in screening. Substantial heterogeneity, small study numbers, and the inability to account for the paired multireader–multicase design limit the strength of these pooled estimates. Until large-scale prospective trials with standardised reporting are available, implementation of AI in lung cancer screening should proceed cautiously, balancing improved sensitivity against workflow disruption and false-positive burden, and these findings should be considered exploratory rather than definitive.

## Figures and Tables

**Table 1:** Summary of the key characteristics of each study.

Study	Year	Country	Study Design	Patients (*n*)	Nodules (*n*)	Readers (*n*)	Reader Experience	AI Type	Primary Outcomes	Additional Outcomes
Bogoni et al. [14]	2012	USA	Retrospective, multi-reader study	43	134	5	Fellowship-trained thoracic radiologists (experience from 1 to 10 years)	Syngo LungCAD integrated into PACS	Sensitivity, False Positives per scan	SR reading time, nodule ≤ 5 mm sensitivity, nodule ≤ 6–10 mm sensitivity
Foti et al. [10]	2010	Italy	Retrospective, multi-reader study	100	258 (≥3 mm)	4	Readers 1–2: 11 and 7 years; Readers 3–4: 4 and 3 years	CAD software (Lung Nodule); version not reported	Sensitivity, False Positives per scan	SR reading time, CR reading time, nodule ≤ 5 mm sensitivity, nodule ≤ 6–10 mm sensitivity
Hsu et al. [15]	2021	Taiwan	Retrospective, multi-reader study	150	340 (222 nodules on conventional CT scans and 118 nodules on LDCT)	6	3 Junior residents: 1–2 years CT, ≥6 months chest CT experience; 3 Senior radiologists with 5, 10, 25 years of experience)	ClearReadCT system with vessel-suppressed and CADe functions	Sensitivity	SR reading time, CR reading time, Junior reader sensitivity, Senior reader sensitivity
Kozuka et al. [16]	2020	Japan	Retrospective, multi-reader study	117	743 (≥3 mm)	2	Two less experienced radiologists (reader A: 5 years, reader B: 1 year of diagnostic experience)	InferRead CT Lung (Infervision)	Sensitivity, False Positives per scan	nodule ≤ 5 mm sensitivity, nodule ≤ 6–10 mm sensitivity, Junior reader sensitivity
Roos et al. [11]	2010	USA	Retrospective, multi-reader study	20	190 (≥3 mm)	3	Three faculty-trained radiologists (9,14 and 24 years of CT experience)	Open CAD system	Sensitivity, False Positives per scan	N/A
Takaishi et al. [17]	2020	Japan	Retrospective, multi-reader study	61	46	3	Radiologists with <10 years of experience (A: 8 years, B: 6 years, C: 2 years)	ClearRead CT	Sensitivity	Junior reader sensitivity
Murchison et al. [18]	2022	United Kingdom	Retrospective, multi-reader study	314 subjects (273 scans in primary analysis)	470 total (269 actionable in primary analysis)	2 (A third reader was present as an adjudicator, but was not a primary reader)	Thoracic Radiologists with ≥9 years of experience	Veye Chest Version 2.0	Sensitivity, False Positives per scan	No reading time data

Abbreviations: AI, artificial intelligence; CAD, computer-aided detection; CADe, computer-aided detection; CR, concurrent reader; CT, computed tomography; LDCT, low-dose computed tomography; *n*, number; N/A, not applicable; PACS, picture archiving and communication system; SR, second reader.

**Table 2:** Summary of the technical characteristics of the included AI systems.

Study	Development Approach	Deep Learning Architecture (If Applicable)	Regulatory Status (If Available)	Intended Clinical Workflow	MIP Reported?
Bogoni et al. [14]	Multi-step algorithm combining computer vision (DGFR) and traditional machine learning (multiple instance learning with gating networks). Trained on 330 datasets and tested on 747 datasets.	Not applicable (the system uses traditional machine learning/classifiers, not a deep learning architecture.	FDA cleared	Second-reader paradigm. The CAD server processes CT data separately, and the resulting CAD findings (DICOM image objects with marked candidates) are automatically sent to PACS for review alongside the routine clinical images.	No
Foti et al. [10]	Software provides automated detection of pulmonary nodules using a dedicated algorithm. After lung segmentation (mean time 45 s), the system highlights nodules on axial images and multiplanar MIP reconstructions. The system allows users to mark nodules missed by the automated detection system and provides morphological/dimensional features (long-axis diameter, area, volume).	Not applicable	-	Can be used as either: (1) Second Reader (SR)—conventional reading first, followed by CAD evaluation of overlooked findings; or (2) Concurrent Reader (CR)—single reading performed simultaneously with CAD prompts.	Yes
Hsu et al. [15]	ClearReadCT system (Riverain Technologies) with vessel-suppressed function and CADe module. Composed of three modules: (1) vessel image removal using a background subtraction algorithm; (2) CADe module that processes lung opacities not subtracted; (3) rendering of boundary contours on detected nodules with report table of density, diameters, and volume.	Not explicitly specified	-	Evaluated in two reading modes: (1) Second-read CAD mode—readers interpret CT images without CAD first, then combine CAD results for final decision; (2) Concurrent-read CAD mode—CAD results simultaneously displayed during initial reading. Intended for both lung cancer screening and clinical routine practice.	No
Kozuka et al. [16]	Deep learning-based CAD system (InferRead CT Lung, Infervision Co., Ltd.) based on a two-stage object detection system derived from the Faster R-CNN framework. Input: CT images after data enhancement (resizing, cropping, padding, flipping). Output: object detection results (bounding boxes and scores). Composed of four parts: Region Proposal Network (RPN), ROI pooling layer, classification, and regression.	Faster R-CNN	-	Second-reader paradigm. Less experienced radiologists read images without CAD first, then with CAD. Designed to assist radiologists; final diagnosis made by physicians.	No
Roos et al. [11]	Traditional feature-based CAD	Not applicable	-	Two-phase interpretation in research setting: (1) Free search without CAD; (2) CAD evaluation of up to 50 CAD detections per patient. CAD list was pre-filtered to remove candidates already identified by readers during free search.	Partial -available as a tool, but it is not stated that MIP was used for all nodule assessment
Takaishi et al. [17]	Computerised vessel-suppression system (ClearReadCT, Riverain Technologies). Automatically subtracts pulmonary vessels from chest CT images while preserving pulmonary nodules.	Not specified	FDA approved and PMDA approved for clinical use	Concurrent-reader mode—radiologists view both computer-aided (vessel-suppressed) CT images and standard CT images simultaneously during interpretation.	No
Murchison et al. [18]	Deep learning-based CAD system (Veye Chest version 2.0, now known as Veye Lung Nodules, Aidence B.V., Amsterdam, the Netherlands). Trained on 45k+ chest CT scans and 40k+ annotations by radiologists. Comprises CADe (detection), CADx (classification), and growth rate calculation functionality.	Deep learning	CE marked and certified as a Class IIb medical device.	Second-reader/assisted reading paradigm in routine clinical population. Radiologists reviewed scans with or without CAD assistance. Software intended for detection, classification, quantification, and growth rate calculation of actionable pulmonary nodules.	No

Abbreviations: CAD, computer-aided detection; CADe, computer-aided detection; CE, Conformité Européenne; CR, concurrent reader; CT, computed tomography; DGFR, difference of Gaussian for ridge detection; DICOM, Digital Imaging and Communications in Medicine; FDA, U.S. Food and Drug Administration; MIP, maximum intensity projection; PACS, picture archiving and communication system; PMDA, Pharmaceuticals and Medical Devices Agency (Japan); R-CNN, region-based convolutional neural network; ROI, region of interest; RPN, region proposal network; SR, second reader.

**Table 3:** Comparison between unaided radiologist and AI-assisted second reader (SR) mode.

Study	Readers	Unassisted Sensitivity	AI-Assisted Sensitivity	Mode
Hsu et al. [15]	6	64%	82%	SR
Roos et al. [11]	3	53%	69%	SR
Foti et al. [10]	2	73.64%	85.66%	SR
Bogoni et al. [14]	5	44%	57%	SR
Kozuka et al. [16]	2	20.9%	38%	SR

**Table 4:** Comparison between unaided radiologist and AI as a concurrent reader (CR).

Study	Readers	Unassisted Sensitivity	AI-Assisted Sensitivity	Mode
Murchison et al. [18]	2	71.9%	80.3%	CR
Takaishi et al. [17]	3	72%	84%	CR
Hsu et al. [15]	6	64%	80%	CR

**Table 5:** False positives per scan reported by included studies. Values are as reported in each primary study. Denominators differed across studies: per reader–case in Bogoni et al. [14], summed across two readers and divided by the number of scans in Foti et al. [10] and Kozuka et al. [16], per patient in Roos et al. [11], and per scan in Murchison et al. [18]. Because of these differences in reporting conventions, values should not be compared directly across studies, and no pooled estimate was calculated.

Study	FP per Scan, Unaided	FP per Scan, AI-Assisted	Direction of Change
Bogoni et al. [14]	0.07	0.15	Increased
Foti et al. [10]	0.56	0.53	Slightly decreased
Kozuka et al. [16]	1.11	2.97	Increased
Roos et al. [11]	1.15	1.45	Increased
Murchison et al. [18]	0.11	0.16	Increased

Abbreviations: AI, artificial intelligence; FP, false positive.

**Table 6:** Summary of secondary outcome meta-analyses. Size thresholds varied across studies. For the ‘≤5 mm’ analysis: Bogoni et al. reported ≥3 mm; Kozuka et al. reported 3–6 mm; Foti et al. reported 3 mm. For the ‘6–10 mm’ analysis: Bogoni et al. reported ≥5 mm; Kozuka et al. reported 6–10 mm; Foti et al. reported 5 mm. These thresholds do not map precisely to the stated intervals. Findings should be interpreted as approximate. Reader experience definitions varied across studies. Kozuka et al. classified readers with 1 and 5 years of experience as ‘junior’; Takaishi et al. reported a combined average for readers with 2, 6, and 8 years of experience; Hsu et al. defined juniors as residents with 1–2 years of CT experience. These definitions are not directly comparable. No included study specified whether nodule size was measured on standard lung parenchymal window images or on MIP reconstructions. Foti et al. [10] used both lung window and multiplanar MIP for viewing but did not specify which was used for size measurement.

Secondary Outcome	Studies (k)	Pooled Effect	95% CI	*p*-Value	Heterogeneity (I^2^)	τ^2^	Q (*p*-Value)
SR reading time difference (seconds)	3	MD = 77.54	(−57.50, 212.6)	0.132	64.9%	1066	5.69 (0.058)
CR reading time difference (seconds)	2	MD = −11.39	(−498.7, 476.0)	0.816	41.3%	1744	1.70 (0.192)
Nodule sensitivity (≤5 mm) (RD)	3	RD = 0.163	(0.074, 0.253)	0.016	22.2%	0.00038	2.59 (0.274)
nodule sensitivity (6–10 mm) (RD)	3	RD = 0.135	(0.073, 0.197)	0.011	0%	0.000	0.77 (0.681)
Junior reader sensitivity (RD)	3	RD = 0.174	(0.033, 0.316)	0.034	52.5%	0.001	4.22 (0.121)
Senior reader sensitivity (RD)	1	RD = 0.11	—	—	—	—	—

Abbreviations: CI, confidence interval; CR, concurrent reader; I^2^, I-squared statistic (heterogeneity); k, number of studies; MD, mean difference; Q, Cochran’s Q statistic; RD, risk difference; SR, second reader; τ^2^, tau-squared (between-study variance).

## Data Availability

The original contributions presented in this study are included in the article/Supplementary Material. Further inquiries can be directed to the corresponding author.

## Supplementary Materials

The following supporting information can be downloaded at: https://www.mdpi.com/article/10.3390/jimaging12100509/s1. Supplementary Table S1: Study-by-outcome data extraction table, including extracted numerators, denominators, reader mode, reader group, reference standard, nodule-size threshold, false-positive definition, and whether each study was included in quantitative or qualitative synthesis. Supplementary Table S2: QUADAS-C risk of bias assessment for the included reader-performance studies. Supplementary Table S3: Study-specific log of all 15 studies excluded at the full-text stage, with reasons for exclusion [55,56,57,58,59,60,61,62,63,64,65]. Supplementary Table S4: Full list of nodule-size thresholds and reader-experience definitions used in each included study, and their mapping to the meta-analysis subgroups. Supplementary Material S1: Full search strategies for PubMed (MEDLINE), Embase, and Cochrane Library, including search dates and syntax. Supplementary Material S2: Full extracted dataset from all included studies, including raw sensitivity, false-positive, and reading-time data, and leave-one-out sensitivity analysis results. Supplementary Material S3: PRISMA 2020 checklist for the systematic review. Supplementary Material S4: PRISMA-DTA checklist for diagnostic test accuracy studies.

## Abbreviations

AI, artificial intelligence; AUC, area under the receiver operating characteristic curve; CAD, computer-aided detection; CADe, computer-aided detection; CADx, computer-aided diagnosis; CE, Conformité Européenne; CI, confidence interval; CLAIM, Checklist for Artificial Intelligence in Medical Imaging; CR, concurrent reader; CT, computed tomography; DGFR, difference of Gaussian for ridge detection; DICOM, Digital Imaging and Communications in Medicine; FDA, U.S. Food and Drug Administration; FN, false negative; FP, false positive; FROC, free-response receiver operating characteristic; HTA, Health Technology Assessment; I^2^, I-squared statistic (heterogeneity); ICC, intraclass correlation coefficient; JAFROC, jackknife free-response receiver operating characteristic; k, number of studies; LDCT, low-dose computed tomography; LLM, large language model; LROC, localization receiver operating characteristic; Lung-RADS, Lung CT Screening Reporting and Data System; MD, mean difference; MDCT, multidetector computed tomography; MIP, maximum intensity projection; MRMC, multi-reader multi-case; N/A, not applicable; NPV, negative predictive value; PACS, picture archiving and communication system; PMDA, Pharmaceuticals and Medical Devices Agency (Japan); PPV, positive predictive value; PRISMA, Preferred Reporting Items for Systematic Reviews and Meta-Analyses; Q, Cochran’s Q statistic; QUADAS-2, Quality Assessment of Diagnostic Accuracy Studies—2; QUADAS-C, Quality Assessment of Diagnostic Accuracy Studies—Comparative; R-CNN, region-based convolutional neural network; RD, risk difference; ROBINS-I, Risk of Bias in Non-randomised Studies of Interventions; ROBVIS, Risk of Bias Visualisation tool; ROI, region of interest; RPN, region proposal network; RR, risk ratio; SD, standard deviation; SR, second reader; STARD-AI, Standards for Reporting of Diagnostic Accuracy Studies—Artificial Intelligence; TP, true positive; TRIPOD+AI, Transparent Reporting of a multivariable prediction model for Individual Prognosis Or Diagnosis—Artificial Intelligence; τ^2^, tau-squared (between-study variance).
