# Supplementary material for: Impact of Artificial Intelligence Assistance on Radiologist Performance in CT-Based Pulmonary Nodule Detection: A Systematic Review and Meta-Analysis of Detection Accuracy Studies
Source: J Imaging. 2026 Oct 14;12(10):509. doi: 10.3390/jimaging12100509 (PMC13706575; doi:10.3390/jimaging12100509)
Supplement: Supplementary file 1 [file jimaging-12-00509-s001.zip › QUADAS-C - Supplementary Table S2.pdf]

QUADAS-C Risk of Bias Assessment

Study	Patient Selection	Index Test	Reference Standard	Flow and Timing	Overall Risk
Bogoni et al. [14]	Low	Low	Moderate[1]	Low	Moderate
Foti et al. [10]	Low	Low	Moderate[1]	Moderate[2]	Moderate
Hsu et al. [15]	Low	Low	Moderate[1]	Low	Moderate
Kozuka et al. [16]	Low	Low	Moderate[1]	Low	Moderate
Roos et al. [11]	Low	Low	Moderate[1]	Low	Moderate
Takaishi et al. [17]	Low	Low	Moderate[1]	Moderate[3]	Moderate
Murchison et al. [18]	Low	Low	Moderate[1]	Low	Moderate

Footnotes:

[1] Reference standard was based on expert consensus rather than histopathological confirmation in most cases; moderate risk.

[2] Potential recall bias due to two-month interval between readings; readers may have remembered findings.

[3] Shorter washout period (15 days) compared to other studies (2 months); potential for recall bias.
