# Supplementary material for: Impact of Artificial Intelligence Assistance on Radiologist Performance in CT-Based Pulmonary Nodule Detection: A Systematic Review and Meta-Analysis of Detection Accuracy Studies
Source: J Imaging. 2026 Oct 14;12(10):509. doi: 10.3390/jimaging12100509 (PMC13706575; doi:10.3390/jimaging12100509)
Supplement: Supplementary file 1 [file jimaging-12-00509-s001.zip › Supplementary Material S1.pdf]

PubMed (MEDLINE)

Search date: [24/03/2026]

Search Syntax:

("artificial intelligence"[Title/Abstract] OR "AI assistance"[Title/Abstract] OR "Deep Learning"[Title/Abstract] OR "computer-assisted"[Title/Abstract] OR "computer aided detection"[Title/Abstract] OR "CAD"[Title/Abstract])
AND
("Radiologist"[Title/Abstract] OR "reader"[Title/Abstract] OR "observer"[Title/Abstract] OR "performance"[Title/Abstract])
AND
("pulmonary nodule"[Title/Abstract] OR "lung nodule"[Title/Abstract] OR "nodule detection"[Title/Abstract])
AND
("computed tomography"[Title/Abstract] OR "chest CT"[Title/Abstract] OR "CT"[Title/Abstract])
AND
("sensitivity"[Title/Abstract] OR "detection accuracy"[Title/Abstract] OR "observer study"[Title/Abstract] OR "reader study"[Title/Abstract] OR "false positive"[Title/Abstract])

Filters applied: None (no date limitations)

Embase

Search date: [24/03/2026]

Search Syntax:

('artificial intelligence'/exp OR 'artificial intelligence' OR 'AI assistance' OR 'deep learning'/exp OR 'deep learning' OR 'computer-assisted' OR 'computer aided detection' OR 'CAD')
AND
('radiologist'/exp OR 'radiologist' OR 'reader' OR 'observer' OR 'performance')
AND
('pulmonary nodule'/exp OR 'pulmonary nodule' OR 'lung nodule' OR 'nodule detection')
AND
('computed tomography'/exp OR 'computed tomography' OR 'chest CT' OR 'CT')
AND
('sensitivity'/exp OR 'sensitivity' OR 'detection accuracy' OR 'observer study' OR 'reader study' OR 'false positive')

Filters applied: None (no date limitations)

Cochrane Library

Search date: [24/03/2026]

Search Syntax:
("artificial intelligence" OR "AI assistance" OR "Deep Learning" OR "computer-assisted" OR "computer aided detection" OR "CAD")
AND
("Radiologist" OR "reader" OR "observer" OR "performance")
AND
("pulmonary nodule" OR "lung nodule" OR "nodule detection")
AND
("computed tomography" OR "chest CT" OR "CT")
AND
("sensitivity" OR "detection accuracy" OR "observer study" OR "reader study" OR "false positive")

Filters applied: None (no date limitations)

NOTE: The categories are reported in the Results section (Section 3.1) and the numerical breakdown is provided in the PRISMA flow diagram (Figure 1)
