# Supplementary material for: Impact of Artificial Intelligence Assistance on Radiologist Performance in CT-Based Pulmonary Nodule Detection: A Systematic Review and Meta-Analysis of Detection Accuracy Studies
Source: J Imaging. 2026 Oct 14;12(10):509. doi: 10.3390/jimaging12100509 (PMC13706575; doi:10.3390/jimaging12100509)
Supplement: Supplementary file 1 [file jimaging-12-00509-s001.zip › Supplementary Material S2.pdf]

Supplementary Material S2: Extracted Dataset

For: Impact of artificial intelligence assistance on radiologist performance in CT-based pulmonary nodule detection: a systematic review and meta-analysis of detection accuracy studies

---

This supplementary material contains all extracted data necessary to reproduce our meta-analyses. The data are presented in tabular format, with values taken directly from the primary studies. Standard deviations were not included as they were not consistently reported across studies.

Contents

1. Extracted Dataset
2. Leave-One-Out Sensitivity Analysis Results
3. Data Dictionary

---

1. Extracted Dataset

Worksheet 1: Study Characteristics

Study	Year	Country	Design	Patients (n)	Nodules (n)	Readers (n)	Reader Experience	AI Type	Reader Mode
Bogoni et al.	2012	USA	Retrospective	43	134	5	1-10 years	Syngo LungCAD	SR
Foti et al.	2010	Italy	Retrospective	100	258	4	3-11 years	Lung Nodule CAD	SR/CR
Hsu et al.	2021	Taiwan	Retrospective	150	340	6	1-25 years	ClearReadCT	SR/CR
Kozuka et al.	2020	Japan	Retrospective	117	743	2	1-5 years	InferRead CT Lung	SR
Roos et al.	2010	USA	Retrospective	20	190	3	9-24 years	Open CAD	SR
Takaishi et al.	2020	Japan	Retrospective	61	46	3	2-8 years	ClearRead CT	CR
Murchison et al.	2022	UK	Retrospective	314	470	2	≥9 years	Veye Chest	CR

Source: Table 1 of main manuscript, derived from primary studies.

Worksheet 2: Sensitivity Data (Primary Analysis)

Study	Total Obs (n)	TP (Unaided)	TP (AI)	Sensitivity (Unaided)	Sensitivity (AI)	RR	RD
Bogoni et al.	670	296	381	44%	57%	1.29	0.13
Foti et al.	516	380	442	73.64%	85.66%	1.16	0.12
Kozuka et al.	1486	310	564	20.9%	38.0%	1.82	0.17

Source:

- Bogoni et al.: Table 1 - Readers 44% (296/670), Reader+CAD 57% (381/670)
- Foti et al.: Table 2 - Reader 1+2 combined: 380/516 unaided, 442/516 with CAD SR
- Kozuka et al.: Table 2 - Total: 310/1486 unaided, 564/1486 with CAD

Pooled Estimates (from RevMan):

- **RR:** 1.39 (95% CI: 1.08 to 1.79), $Z = 2.56$, $p = 0.01$, $I^2 = 95\%$
- **RD:** 0.14 (95% CI: 0.11 to 0.18), $Z = 8.10$, $p < 0.0001$, $I^2 = 47\%$

Worksheet 3: False Positives Per Scan

These values are presented narratively in the main manuscript (Table 5). They were not pooled quantitatively because four of five studies did not report standard deviations and the studies were heterogeneous in populations, CAD systems, and false-positive definitions. Imputation using coefficients of variation from a single study was considered but rejected as not defensible. FP, false positives per scan. Values are as reported in each primary study. Denominators differed across studies: per reader–case in Bogoni et al., summed across two readers and divided by number of scans in Foti et al. and Kozuka et al., per patient in Roos et al., and per scan in Murchison et al. Because of these differences, values should not be compared directly across studies, and no pooled estimate was calculated.

Study	Mean FP (Unaided)	Mean FP (AI)	Total
Bogoni et al.	0.07	0.15	43
Foti et al.	0.56	0.53	100
Kozuka et al.	1.11	2.97	117
Murchison et al.	0.11	0.16	273
Roos et al.	1.15	1.45	20

Source:

- Bogoni et al.: Results - "0.07 (95% CI, ±0.04)" and "0.15 (95% CI, ±0.08)"
- Foti et al.: Table 2 - FP counts converted to per scan (56/100 = 0.56; 53/100 = 0.53)

- Kozuka et al.: Table 2 - FP counts converted to per scan (130/117 = 1.11; 348/117 = 2.97)
- Roos et al.: Results - "1.15 FP/patient" and "1.45 FP/patient"
- Murchison et al.: Results - "0.11" and "0.16"

Worksheet 4a: Reading Time – SR Mode (seconds)

Study	Mean (Unaided)	Mean (AI)	MD	95% CI	Weight
Bogoni et al.	143	225	82	[57.62, 106.38]	50.12%
Foti et al.	135	330	195	[58.86, 331.14]	10.39%
Hsu et al.	156	197	41	[-2.08, 84.08]	39.49%

Source:

- Bogoni et al.: Results - 2m23s (143s) unaided; 3m45s (225s) with CAD
- Foti et al.: Table 3 - Average 135s unaided; 330s with CAD SR
- Hsu et al.: Table 3 - All readers average 156s unaided; 197s with CAD SR

Pooled Estimate (from JASP):

- **MD:** 77.54 (95% CI: -57.50 to 212.6), $p = 0.132$, $I^2 = 64.9\%$, $\tau^2 = 1066$

Worksheet 4b: Reading Time – CR Mode (seconds)

Study	Mean (Unaided)	Mean (AI)	MD	95% CI	Weight
Foti et al.	135	195	60	[-76.14, 196.14]	22.40%
Hsu et al.	156	124	-32	[-56.17, -7.83]	77.60%

Source:

- Foti et al.: Table 3 - 135s unaided; 195s with CAD CR
- Hsu et al.: Table 3 - 156s unaided; 124s with CAD CR

Pooled Estimate (from JASP):

- **MD:** -11.39 (95% CI: -498.7 to 476.0), $p = 0.816$, $I^2 = 41.3\%$

Worksheet 4c: Nodule Size Sensitivity (RD)

Nodules ≤5 mm (Exploratory):

Study	RD	95% CI	Weight
Bogoni et al.	0.13	[0.04, 0.22]	18.23%
Foti et al.	0.12	[0.04, 0.21]	21.18%
Kozuka et al.	0.19	[0.15, 0.23]	60.59%

Source:

- Bogoni et al.: Table 1 - ≥3mm: 44% to 57% (RD=0.13)
- Foti et al.: Table 2 - 3mm threshold: 73.64% to 85.66% (RD=0.12)
- Kozuka et al.: Table 2 - 3-6mm: 13.7% to 32.4% (RD=0.187)

Pooled Estimate (from JASP):

- **RD:** 0.163 (95% CI: 0.074 to 0.253), $p = 0.016$, $I^2 = 22.2\%$, $\tau^2 = 0.0003784$

Nodules 6-10 mm:

Study	RD	95% CI	Weight
Bogoni et al.	0.16	[0.07, 0.25]	27.84%
Foti et al.	0.11	[0.04, 0.18]	41.60%
Kozuka et al.	0.14	[0.06, 0.23]	30.56%

Source:

- Bogoni et al.: Table 1 - ≥5mm: 44% to 60% (RD=0.16)
- Foti et al.: Table 2 - 5mm threshold: 77.90% to 87.94% (RD=0.10)
- Kozuka et al.: Table 2 - 6-10mm: 33.3% to 47.6% (RD=0.143)

Pooled Estimate (from JASP):

- **RD:** 0.135 (95% CI: 0.073 to 0.197), $p = 0.011$, $I^2 = 0\%$

Worksheet 4d: Reader Experience Sensitivity (RD)

Study	RD	95% CI	Weight
Hsu et al.	0.27	[0.15, 0.35]	22.75%
Kozuka et al.	0.17	[0.13, 0.21]	48.38%
Takaishi et al.	0.12	[0.04, 0.20]	28.87%

Source:

- Hsu et al.: Table 2 - Junior: 52% to 79% (SR, RD=0.27)
- Kozuka et al.: Table 2 - Combined junior: 20.9% to 38.0% (RD=0.17)
- Takaishi et al.: Table 2 - Average: 72% to 84% (RD=0.12)

Pooled Estimate (from JASP):

- **RD:** 0.174 (95% CI: 0.033 to 0.316), $p = 0.034$, $I^2 = 52.5\%$, $\tau^2 = 0.001$

Worksheet 5: Publication Bias Analysis (Sensitivity) – From JASP Output

Test	Estimate	SE	t	df	p	95% CI Lower	95% CI Upper
PET (Egger's)	0.568	0.483	1.176	1	0.449	-0.378	1.514
PEESE	0.924	0.246	3.757	1	0.166	0.442	1.405

Source: JASP output from publication bias analysis (PET-PEESE)

---

2. Leave-One-Out Sensitivity Analysis Results – From JASP

2.1 SR Reading Time (MD)

Study Excluded	Pooled MD
None (Original)	77.54
Bogoni et al.	95.94
Foti et al.	22.84
Hsu et al.	62.41

Source: JASP Casewise Diagnostics table from SR Reading Time analysis.

2.2 Nodules ≤5 mm (RD)

Study Excluded	Pooled RD
None (Original)	0.163
Bogoni et al.	0.029
Foti et al.	0.018
Kozuka et al.	0.000

Source: JASP Casewise Diagnostics table from small nodule sensitivity analysis.

2.3 Nodules 6-10 mm (RD)

Study Excluded	Pooled RD
None (Original)	0.135
Bogoni et al.	0.000
Foti et al.	0.000
Kozuka et al.	0.000

Source: JASP Casewise Diagnostics table from medium nodule sensitivity analysis.

Note: The 'Leave One Out' value of 0.000 for all studies indicates that removing any single study leaves only two studies, which is insufficient for the random-effects model to estimate τ^2, resulting in a pooled estimate of zero.

2.4 Junior Reader Sensitivity (RD)

Study Excluded	Pooled RD
None (Original)	0.174
Hsu et al.	0.017
Kozuka et al.	0.080
Takaishi et al.	0.041

Source: JASP Casewise Diagnostics table from junior reader sensitivity analysis.

---

3. Data Dictionary

Variable	Definition
TP	True Positives (number of nodules correctly detected)

Variable	Definition
FP	False Positives (per scan or per case)
Sensitivity	TP / (TP + FN)
RR	Risk Ratio = Sensitivity_AI / Sensitivity_Unaided
RD	Risk Difference = Sensitivity_AI - Sensitivity_Unaided
MD	Mean Difference = Mean_AI - Mean_Unaided
SE	Standard Error
CI	Confidence Interval
SR	Second Reader Mode
CR	Concurrent Reader Mode
