# Supplementary material for: Impact of Artificial Intelligence Assistance on Radiologist Performance in CT-Based Pulmonary Nodule Detection: A Systematic Review and Meta-Analysis of Detection Accuracy Studies
Source: J Imaging. 2026 Oct 14;12(10):509. doi: 10.3390/jimaging12100509 (PMC13706575; doi:10.3390/jimaging12100509)
Supplement: Supplementary file 1 [file jimaging-12-00509-s001.zip › Supplementary Table S1.pdf]

Study	Outcome	Reader Mode	Reader Group	Reference Standard	Nodule Size Threshold	FP Definition	Numerator (AI)	Denominator (AI)	Numerator (Unaided)	Denominator (Unaided)	Included in Meta-analysis?
Bogoni et al. [14]	Sensitvity	SR	All	Expert Consensus	≥ 3 mm	-	381	670	296	670	Yes
Bogoni et al. [14]	False positives	SR	All	Expert consensus	≥ 3 mm	Marks not associated with true nodules (per-case FP rate)	Not reported as a raw count – only mean (0.15 per case)	Not reported	Not reported – only mean (0.07 per case) given.	Not reported.	Yes – Narratively
Foti et al. [10]	Sensitivity	SR	All	Expert consensus	≥ 3 mm (Primary)	-	442	516	380	516	Yes
Foti et al. [10]	False positives	SR	All	Expert consensus	≥ 3 mm (Primary)	CAD marks not corresponding to true nodules (per-scan FP rate)	Not reported – only total FPs per reader are given in Table 2 (23 and 30 for SR readers), but these are total FPs across all cases, not per scan counts with standard deviations.	Not reported – the denominator for FP/scan would be the number of scans (100), but the paper reports total FPs without linking them to per-scan variance data.	Not reported – Table 2 shows Reader 1 (unaided) FP = 25; Reader 2 (unaided) FP = 31. Total = 56. Again, no per-scan variance data.	Not reported	Yes – Narratively
Foti et al. [10]	Reading time	SR + CR	All	Expert consensus	≥ 3 mm	-	-	-	-	-	Yes – included in both SR and CR reading time secondary analyses.
Hsu et al. [15]	Sensitivity	SR + CR	Junior + Senior	Radiology reports + Consensus Panel	≤10 mm	-	Not reported (percentages only: 82% SR, 80% CR)	Not reported	Not reported (64%)	Not reported	No (qualitative synthesis only)
Hsu et al. [15]	Reading time	SR + CR	Junior + Senior	Radiology reports + Consensus Panel	≤10 mm	-	N/A (continuous outcome; means: SR = 197s, CR = 124s; SDs available)	N/A	N/A (mean = 156s; SD available)	N/A	Yes
Kozuka et al. [16]	Sensitivity	SR	Junior	Radiology reports + Expert Consensus Panel	≥3 mm	-	564	1486	310	1486	Yes

Kozuka et al. [16]	False positives	SR	Junior	Radiology reports + Expert Consensus Panel	≥3 mm	Marks not corresponding to true nodules	Not reported (total FPs = 348; per-scan mean with SD not provided)	Not reported	Not reported (total FPs = 130; per-scan mean with SD not provided)	Not reported	Yes – Narratively
Roos et al. [11]	Sensitivity	SR	Senior	Expert panel	≥3 mm	-	Not reported (mean sensitivity = 69%; range: 60–82%)	Not reported	Not reported (mean sensitivity = 53%; range: 44–59%)	Not Reported	No (qualitative synthesis only)
Roos et al. [11]	False positives	SR	Senior	Expert panel	≥ 3 mm	Marks not corresponding to true nodules	Not reported (mean FP/patient = 1.45; range: 0.85–2.7)	Not reported	Not reported (mean FP/patient = 1.15; range: 0.6–2.1)	Not reported	Yes - Narratively
Takaishi et al. [17]	Sensitivity	CR	Junior	Expert panel	≥4 mm	-	Not reported (mean sensitivity = 84%; range: 80–89%)	Not reported	Not reported (mean sensitivity = 72%; range: 61–78%)	Not reported	No (qualitative synthesis only)
Murchison et al. [18]	Sensitivity	CR	Senior	Majority consensus (with third reader adjudication)	Actionable nodules ≥5 mm (or volume ≥80 mm³) and ≤30 mm	-	Not reported (mean sensitivity = 80.3%; 95% CI: 75.2%, 85.0%)	Not reported	Not reported (mean sensitivity = 71.9%; 95% CI: 66.0%, 77.0%)	Not reported	No (qualitative synthesis only)
Murchison et al. [18]	False positives	CR	Senior	Majority consensus	Actionable nodules ≥5 mm (or volume ≥80 mm³) and ≤30 mm	CAD prompts not corresponding to actionable nodules in the reference standard	Not reported (mean FP/scan = 0.16)	Not reported	Not reported (mean FP/scan = 0.11)	Not reported	Yes - Narratively
