# Supplementary material for: Impact of Artificial Intelligence Assistance on Radiologist Performance in CT-Based Pulmonary Nodule Detection: A Systematic Review and Meta-Analysis of Detection Accuracy Studies
Source: J Imaging. 2026 Oct 14;12(10):509. doi: 10.3390/jimaging12100509 (PMC13706575; doi:10.3390/jimaging12100509)
Supplement: Supplementary file 1 [file jimaging-12-00509-s001.zip › Supplementary Table S3.pdf]

	Study (first, year,author)	Reason for exclusion	Category
1	Lo et al., 2018	Study evaluated a vessel-suppressed CADe (VIS/CADe) system, which combines pulmonary vessel subtraction with computer-aided detection. The intervention is not a standard AI-assisted (second- or concurrent-reader) paradigm but a novel vessel-suppression display. The primary outcomes are LROC-AUC, sensitivity, specificity, PPV, and NPV — no extractable false positives per scan in a form poolable with other included	No extractable paired sensitivity/FP data; non-standard AI-assisted reading paradigm (vessel suppression)
2	Morozov et al., 2021	Study describes the development of a cluster model and annotation tool for creating a collaborative dataset of lung cancer CT scans. No AI-assisted vs unaided reader comparison, no detection accuracy outcomes, no reading time data. The paper is about dataset creation methodology, not AI-assisted detection performance.	Not a primary AI-assisted detection study (dataset/tool development only)
3	Liu J, 2020	Evaluates CAD for malignancy classification of pulmonary nodules; outcomes are AUC,	Diagnostic parameters rather than detection

		sensitivity, specificity for benign vs malignant, not nodule detection sensitivity or FP per scan.	
4	Chao et al., 2023	Study compared AI-assisted vs unaided reading using a paired MRMC design, but reported sensitivity and specificity only as pooled averages (means ± SD) rather than extractable per-nodule TP/FP/FN counts. Additionally, nodule size subgroups (<5 mm, 6–10 mm, 11–20 mm, >20 mm) do not align with the size thresholds used in this meta-analysis (≤5 mm, 6–10 mm). No 2×2 data available for pooling.	No extractable sensitivity or false positive data
5	Kim RY, 2022	Evaluates AI-based CAD for malignancy risk stratification and management of already-detected indeterminate pulmonary nodules; outcomes are AUC, sensitivity/specificity at malignancy-risk thresholds, and interobserver agreement, not nodule detection sensitivity or FP per scan.	Diagnostic/classification rather than detection; no extractable detection sensitivity/FP data
6	Hwang et al., 2026	Prospective randomized controlled trial comparing AI-assisted vs unaided LDCT interpretation. However, the study reported interpretation time (primary outcome) and detection rates (proportion of examinations reporting a finding) rather than sensitivity	No extractable sensitivity or false positive data

		(TP/(TP+FN)) or false positives per scan. No 2×2 data, no reference standard for nodule-level truth, and the outcome is measured at the	
7	Das et al., 2006	Study compared two CAD systems (ImageChecker CT and NEV) in a paired reader design, but the reference standard and the unit of analysis (per-nodule detection of nodules as small as 1–2 mm) are not comparable to the other included studies. The mean nodule diameter was only 3.4 mm, with 89/116 (77%) of nodules <5 mm — a size range not used in clinical decision-making. Additionally, no extractable 2×2 data, SDs, or per-reader TP/FP/FN counts were reported.	No extractable sensitivity or false positive data
8	White CS et al, 2008	Multicentre CAD second-reader study; outcomes reported as AUC and actionability ratings, not nodule-level sensitivity or false positives per scan.	No extractable sensitivity/FP data; diagnostic parameters not detection
9	Li X, 2019	Stand-alone AI vs radiologist comparison; no AI-assisted vs unaided radiologist reading.	No AI-assisted vs unaided comparison (standalone AI only)
10	Way T, 2010	Evaluates CAD effect on radiologists' likelihood-of-malignancy estimates for lung nodules; outcomes are ROC/AUC for malignancy, not nodule detection sensitivity or false positives per scan.	Diagnostic parameters rather than detection; no extractable detection sensitivity/FP data
11	Lan et al., 2022	Study compared AI-assisted vs unaided reading, but reported only sensitivity and false positives per scan (overall and by location, size,	No extractable non-sensitivity/FP data; unpaired reader design

		and texture subgroups). No reading time, AUC/FOM, PPV/NPV, or reader confidence data were reported. Additionally, the design compared two separate reader groups (2 without AI vs 3 with AI) rather than a paired within-reader comparison, and used a consensus reference standard rather than a nodule-level gold standard. No extractable non-sensitivity/non-FP outcomes for pooling.	
12	Wataya T, 2022	Evaluates characterization and diagnosis (malignancy) of pulmonary nodules/masses with and without DL-based CAD; outcomes are AUC for characteristics, ICC, and assessment time, not nodule detection sensitivity or FP per scan.	Diagnostic parameters rather than detection; no detection sensitivity/FP data
13	Marten et al., 2005	Study evaluated CAD stand-alone and reader performance for pulmonary nodule detection, but the comparison was against an independent gold standard rather than a paired AI-assisted vs unaided within-reader design for sensitivity or FP per scan. Outcomes reported were detection rates, FP per examination, and ROC/AUC by nodule subgroup (size, margin, attachment) — no extractable 2×2 data or SDs for continuous outcomes, and no standard AI-assisted (second- or concurrent-reader) paradigm for the same reader group.	Detection,No extractable paired AI-assisted sensitivity/FP data; non-standard AI-assisted reading paradigm
14	Shah SK, 2005	Evaluates CAD output for benign vs malignant diagnosis of solitary pulmonary nodules;	Diagnostic parameters rather than detection; no

		outcome is AUC for diagnosis, not nodule detection.	detection sensitivity/FP data
15	Hempel et al., 2022	Study evaluated the impact of CAD on pulmonary nodule management recommendations (BTS grades) and interobserver agreement. Primary outcomes were management recommendation grades and kappa agreement, not nodule-level sensitivity or false positives per scan. Reading time was reported (unaided vs aided), but the study used a management recommendation endpoint rather than nodule detection accuracy, and no extractable 2×2 data or SDs for detection outcomes were provided.	Detection: No extractable sensitivity or false positive data; management recommendation endpoint

Bibliography

55. Lo SB, Freedman MT, Gillis LB, White CS, Mun SK. Computer-aided detection of lung nodules on CT with a computerized pulmonary vessel suppressed function. *AJR Am J Roentgenol.* 2018;210(3):480–488. doi:10.2214/AJR.17.18718
56. Morozov SP, Gombolevskiy VA, Elizarov AB, et al. A simplified cluster model and a tool adapted for collaborative labeling of lung cancer CT scans. *Comput Methods Programs Biomed.* 2021;206:106111. doi:10.1016/j.cmpb.2021.106111
57. Liu J, Zhao L, Han X, Ji H, Liu L, He W. Estimation of malignancy of pulmonary nodules at CT scans: effect of computer-aided diagnosis on diagnostic performance of radiologists. *Asia Pac J Clin Oncol.* 2020;16(6):e199–e204. doi:10.1111/ajco.13362
58. Chao HS, Tsai CY, Chou CW, et al. Artificial intelligence assisted computational tomographic detection of lung nodules for prognostic cancer examination: a large-scale clinical trial. *Biomedicines.* 2023;11(1):147. doi:10.3390/biomedicines11010147
59. Kim RY, Oke JL, Pickup LC, et al. Artificial intelligence tool for assessment of indeterminate pulmonary nodules detected with CT. *Radiology.* 2022;304(3):683–691. doi:10.1148/radiol.212182
60. Hwang EJ, Lee T, Lim WH, et al. Artificial intelligence-assisted lung nodule evaluation on low-dose chest CT in asymptomatic individuals: a prospective

randomized controlled trial. *AJR Am J Roentgenol.* 2026;226(3):1–12. doi:10.2214/AJR.26.34552
61. Das M, Mühlenbruch G, Mahnken AH, et al. Small pulmonary nodules: effect of two computer-aided detection systems on radiologist performance. *Radiology.* 2006;241(2):564–571. doi:10.1148/radiol.2412051139
62. White CS, Pugatch R, Koonce T, Rust SW, Dharaiya E. Lung nodule CAD software as a second reader: a multicenter study. *Acad Radiol.* 2008;15(3):326–333. doi:10.1016/j.acra.2007.09.014
63. Li L, Liu Z, Huang H, Lin M, Luo D. Evaluating the performance of a deep learning-based computer-aided diagnosis (DL-CAD) system for detecting and characterizing lung nodules: comparison with the performance of double reading by radiologists. *Thorac Cancer.* 2019;10(2):183–192. doi:10.1111/1759-7714.12931
64. Way T, Chan HP, Hadjiiski L, et al. Computer-aided diagnosis of lung nodules on CT scans: ROC study of its effect on radiologists' performance. *Acad Radiol.* 2010;17(3):323–332. doi:10.1016/j.acra.2009.10.016
65. Lan CC, Hsieh MS, Hsiao JK, et al. Deep learning-based artificial intelligence improves accuracy of error-prone lung nodules. *Int J Med Sci.* 2022;19(3):490–498. doi:10.7150/ijms.69400
66. Wataya T, Yanagawa M, Tsubamoto M, et al. Radiologists with and without deep learning-based computer-aided diagnosis: comparison of performance and interobserver agreement for characterizing and diagnosing pulmonary nodules/masses. *Eur Radiol.* 2023;33(1):348–359. doi:10.1007/s00330-022-08948-4
67. Marten K, Engelke C, Seyfarth T, Grillhösl A, Obenauer S, Rummeny EJ. Computer-aided detection of pulmonary nodules: influence of nodule characteristics on detection performance. *Clin Radiol.* 2005;60(2):196–206. doi:10.1016/j.crad.2004.05.012
68. Shah SK, McNitt-Gray MF, De Zoysa KR, et al. Solitary pulmonary nodule diagnosis on CT: results of an observer study. *Acad Radiol.* 2005;12(4):496–501. doi:10.1016/j.acra.2004.11.024
69. Hempel HL, Engbersen MP, Wakkie J, van Kelckhoven BJ, de Monyé W. Higher agreement between readers with deep learning CAD software for reporting pulmonary nodules on CT. *Eur J Radiol Open.* 2022;9:100435. doi:10.1016/j.ejro.2022.100435
