# Supplementary material for: Impact of Artificial Intelligence Assistance on Radiologist Performance in CT-Based Pulmonary Nodule Detection: A Systematic Review and Meta-Analysis of Detection Accuracy Studies
Source: J Imaging. 2026 Oct 14;12(10):509. doi: 10.3390/jimaging12100509 (PMC13706575; doi:10.3390/jimaging12100509)
Supplement: Supplementary file 1 [file jimaging-12-00509-s001.zip › Supplementary Table S4.pdf]

Supplementary Table S4

Reported nodule-size thresholds in included studies and their mapping to the meta-analysis subgroups

This table documents the exact nodule-size thresholds reported in each included study and how they were mapped to the meta-analysis subgroups (≤5 mm and 6–10 mm). Because the thresholds are not equivalent across studies, the pooled estimates from these subgroup analyses should be interpreted as approximate and exploratory only. All studies in the "≤5 mm" analysis included nodules ≤5 mm, and all studies in the "6–10 mm" analysis included nodules within the 6–10 mm range; however, the exact boundaries differ. Future studies should adopt standardised size thresholds to facilitate pooling.

Study (first author, year)	AI/CAD system	Reported threshold(s) in original study	Mapped to meta-analysis subgroup	Equivalent to target category?	Notes
**Bogoni 2012**	CAD (PACS-integrated)	≥3 mm	≤5 mm	No	Threshold includes nodules <3 mm
**Bogoni 2012**	CAD (PACS-integrated)	≥5 mm	6–10 mm	No	Threshold includes nodules 5–6 mm
**Foti 2010**	CAD	3 mm threshold	≤5 mm	No	Threshold is below 5 mm
**Foti 2010**	CAD	5 mm threshold	6–10 mm	No	Threshold is below 6 mm
**Hsu 2021**	ClearRead CT (Riverain)	<3 mm; 3–6 mm; 6–10 mm	≤5 mm; 6–10 mm	Partially	3–6 mm straddles both subgroups; 6–10 mm is an exact match
**Hu 2022**	InferRead CT Lung (Infervision)	≤4 mm; >4 to <6 mm; 6–8 mm; >8 mm	≤5 mm; 6–10 mm	No	None of the categories align exactly with ≤5 mm or 6–10 mm
**Kozuka 2020**	InferRead CT Lung (Infervision)	3–6 mm; 6–10 mm	≤5 mm; 6–10 mm	Partially	3–6 mm extends to 6 mm; 6–10 mm is an exact match
